# Head-to-Head Comparison of Sirolimus-Eluting Stents versus Paclitaxel-Eluting Stents in Patients Undergoing Percutaneous Coronary Intervention: A Meta-Analysis of 76 Studies

**DOI:** 10.1371/journal.pone.0097934

**Published:** 2014-05-20

**Authors:** Xinlin Zhang, Jun Xie, Guannan Li, Qinhua Chen, Biao Xu

**Affiliations:** Department of Cardiology, Affiliated Drum Tower Hospital, Nanjing University Medical School, Nanjing, Jiangsu, China; University of Hull, United Kingdom

## Abstract

**Background:**

The relative short-, long- and overall-term efficacy and safety of sirolimus-eluting stents (SES, Cypher) compared with paclitaxel-eluting stents (PES, Taxus) in large head-to-head comparisons still remain to be defined.

**Methods:**

We searched Pubmed, EMBASE, and the Cochrane Central Register of Controlled Trials (CENTRAL) for articles comparing outcomes of interest between SES and PES without language restriction. Short- (≤1 year), long- (>1 year), and overall-term (the longest follow-up of each study) outcomes were evaluated. The primary endpoint was target lesion revascularization (TLR). Other outcomes of interest were target vessel revascularization (TVR), myocardial infarction, all-cause death, cardiac death, stent thrombosis, major adverse cardiac events (MACEs), restenosis and late lumen loss.

**Results:**

Seventy-six studies including more than 15000 patients in randomized controlled trials and over 70000 patients in adjusted observational studies were included. At overall-term follow-up, SES significantly reduced TLR (relative risk [RR]: 0.61; 95% confidence interval [CI]: 0.49–0.76), TVR (RR: 0.67; 95% CI: 0.54–0.83), MACE (RR: 0.79; 95% CI: 0.72–0.87), myocardial infarction (RR: 0.85; 95% CI: 0.73–0.99), in-segment restenosis (RR: 0.50; 95% CI: 0.38–0.65), and in-segment late lumen loss (weighted mean difference [WMD]: −0.19; 95% CI: −0.24–−0.14) in randomized controlled trials compared with PES. In addition, lower rates of death (RR: 0.91; 95% CI: 0.83–1.00), any stent thrombosis (RR: 0.62; 95% CI: 0.45–0.86), definite stent thrombosis (RR: 0.59; 95% CI: 0.45–0.77) were found in patients receiving SES in adjusted observational studies. Largely similar results were found at short- and long-term follow-up, and in patients with diabetes, acute myocardial infarction or long lesions.

**Conclusions:**

SES significantly reduced the short-, long- and overall-term risk of TLR/TVR, MACE, and restenosis, and overall-term risk of myocardial infarction in randomized controlled trials, as compared with PES. Lower rates of death and stent thrombosis were also observed in observational studies in SES-treated patients.

## Introduction

Percutaneous coronary interventions (PCIs) are being widely used in patients with coronary artery disease. Bare-metal stents (BMS) can dramatically reduce the risk of re-occlusion and re-infarction after PCIs [1], but are always associated with an increased risk of restenosis [2]. The emergence of drug-eluting stents (DES), in which sirolimus-eluting stents (SES) and paclitaxel-eluting stents (PES) are two of the most extensively studied, has largely solved this problem: they reduce the rate of angiographic restenosis and the need for re-intervention [3]–[5]. Several prior meta-analyses have been carried out to evaluate the relative performance of SES and PES [6]–[8]. However, almost all these analyses were conducted based on randomized controlled trials, either without a long-term follow-up or conducting indirect comparisons. It is generally accepted that well designed head-to-head studies, especially randomized controlled trials, provide the most rigorous evidence, and large-scale observational studies might provide more power to detect differences in low-frequency events. Herein, by employing all the head-to-head studies available, we performed a meta-analysis of head-to-head comparison of SES with PES in patients undergoing PCIs in randomized controlled trials, adjusted and non-adjusted observational studies at short-, long-, and overall-term follow-up. In addition, we performed analyses in populations with diabetes mellitus, acute myocardial infarction, and long coronary lesions.

## Materials and Methods

### Search strategy and Selection criteria

We searched Pubmed, EMBASE, and the Cochrane Central Register of Controlled Trials (CENTRAL) from their inception until March 26, 2013 using the key words (‘percutaneous coronary intervention’ OR PCI) AND (sirolimus OR rapamycin OR SES OR Cypher) AND (paclitaxel OR PES OR Taxus) or variants of these terms. References of selected articles and reviews were manually reviewed for potential relevant citations. No language, publication status restrictions were imposed.

To be included in the meta-analysis, studies have to make head-to-head comparisons of SES (Cypher or Cypher Select, Cordis, Miami Lakes, FL, USA) with PES (Taxus Express/Liberte, Boston Scientific, Natick, MA, USA) in patients undergoing percutaneous coronary intervention; the lengths of follow-up were at least 6 months, and all studies reported the outcomes of interest (they either provided original data necessary to calculate relative risk or directly provided relative risks with confidence intervals). All randomized controlled trials and observational studies that fulfilled the inclusion criteria were included. We excluded studies that used “non-Cypher” SES and “non-Taxus” PES. The meta-analysis was conducted and reported in accordance with the PRISMA (Preferred Reporting Items for Systematic Reviews and Meta-Analyses) checklist (**File S1**) [9].

### Data collection and Assessment of quality

Two investigators (X.L.Z. and J.X.) independently reviewed the titles/abstracts, assessed study eligibility, and extracted the data. Disagreements were resolved by consensus. The following data were extracted from each eligible study: name of trial/registry/first author, year of publication, patient profile, number of patients, age, characteristics of the population (gender, diabetes mellitus, hypertension, hyperlipidemia, prior myocardial infarction, acute coronary syndrome), and duration of follow-up. For randomized controlled trials and non-adjusted observational studies, the absolute numbers of events were extracted for the measures of risk. In adjusted observational studies (propensity matching, covariate adjustment, or propensity-based adjustment), hazard ratios (relative risks) and their corresponding 95% confidence intervals (95% CIs) were directly extracted. The bias risk of randomized controlled trials was assessed by evaluating the following methodological criteria recommended by Cochrane Collaboration: sequence generation of the allocation, concealment of allocation, blinding, incomplete outcome data, selective outcome reporting, and other sources of bias [10]. Trials fulfilled the first 3 components were considered as studies with low risk of bias. Jadad scale was also used to score the included randomized controlled trials [11]. The quality of observational studies was assessed by the Newcastle-Ottawa Scale criteria [12].

### Outcomes

Short- (≤1 year), long- (>1 year) and the overall-term (the longest follow-up of each study) efficacy and safety outcomes were evaluated. The primary outcome of interest was target lesion revascularization (TLR), which was defined as any revascularization procedure, percutaneous or surgical, involving the target lesion. Other clinical endpoints were myocardial infarction (MI), death, cardiac death, stent thrombosis, target vessel revascularization (TVR), the composite of death and myocardial infarction, and major adverse cardiac events (MACEs). MACE was generally defined as the composite of death, myocardial infarction, and TLR; several studies reported cardiac death or TVR in MACE, and were used as proxy measures. The angiographic outcomes were in-stent restenosis, in-segment restenosis, in-stent late lumen loss (LLL), and in-segment late lumen loss. The definitions of angiographic outcomes were described elsewhere [13].

### Statistical analysis

The relative risks (RRs) and 95% confidence intervals (CIs) were used as the summary statistics for binary variables, while weighted mean differences (WMDs) and 95% CIs were effect estimates for continuous variables. Hazard ratios (HRs) and odds ratios (ORs) were directly considered as RRs. Given the limitations of considering ORs as RRs, we performed a sensitivity analysis that excluded the 5 observational studies which reported ORs. For studies in which only 1 of the arms had no event of interest, the treatment effect estimate and its confidence interval were computed after adding 0.5 to each cell of the 2×2 table [14]. The RR and WMD in each study was pooled using the random model (DerSimonian and Laird method) [15] or the fixed model (Mantel-Haenszel method) [16] according to the heterogeneity across studies. Heterogeneity was evaluated with the χ2-based Q test [17], and a p cut-off value of 0.10 suggests significant heterogeneity. Meanwhile, we used the *I^2^* index to measure the consistency between studies, with values <25%, 25–50%, >50% indicating low, moderate, and high heterogeneity respectively [18]. Publication bias was assessed by visually inspecting the funnel plots and by performing Begg's test, and a p<0.05 was considered as the existence of significant publication bias [19]. Sensitivity analyses were conducted in the following groups: studies with a low risk of bias, and studies with the duration of dual antiplatelet therapy for at least 6 months [20] to evaluate the consistency of our main findings. Meanwhile, we performed meta-analyses of main outcomes in subgroups of patients with diabetes mellitus, acute myocardial infarction and long coronary lesions. All the statistical analyses were carried out using the STATA version 11.0 (STATA Corporation, College Station, TX, USA) software.

## Results

### Eligible studies

**Figure 1:**
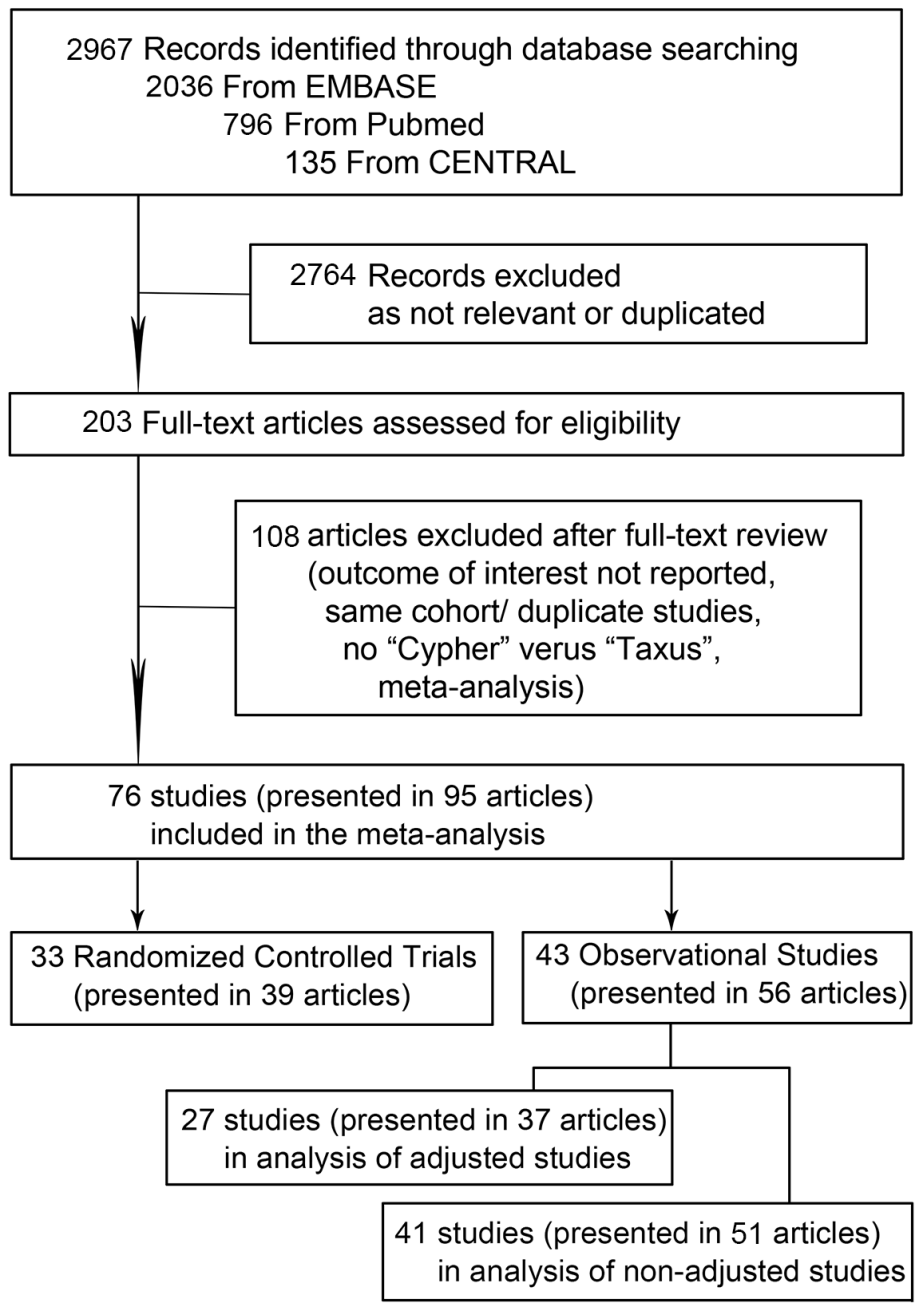
Flow Diagram of Meta-Analysis.

The flow diagram of the study is shown in **Figure 1**. Of the 2967 potentially relevant articles initially screened, 95 articles involving 76 studies met our inclusion criteria and were included in the meta-analysis. Among them, 33 studies (7590 persons in SES arm, 7520 in PES) presented in 39 articles were randomized controlled trials, 27 studies (39904 persons in SES arm, 31694 in PES) presented in 37 articles were adjusted observational studies, and 41 studies (44734 persons in SES arm, 33240 in PES) presented in 51 articles were non-adjusted observational studies. 25 studies presented in 32 articles reported both adjusted and non-adjusted results of outcomes. Baseline characteristics of the patients enrolled in the studies and the length of clinical follow-up are shown in **Table S1**. The mean age ranged from 53 to 69 years, the percentage of male from 55% to 91%, and the percentage of patients with diabetes from 0 to 100%. The length of follow-up ranged from to 6 to 60 months. The recommended duration of clopidogrel therapy was at least 6 months in most studies, while 2 randomized controlled trials and 9 observational studies failed to report the length of dual antiplatelet therapy. The references of the 95 articles representing 76 studies included in the meta-analysis were listed in appendix **File S2**. The quality scales of these studies were shown in **File S3**.

### Target lesion revascularization

**Figure 2:**
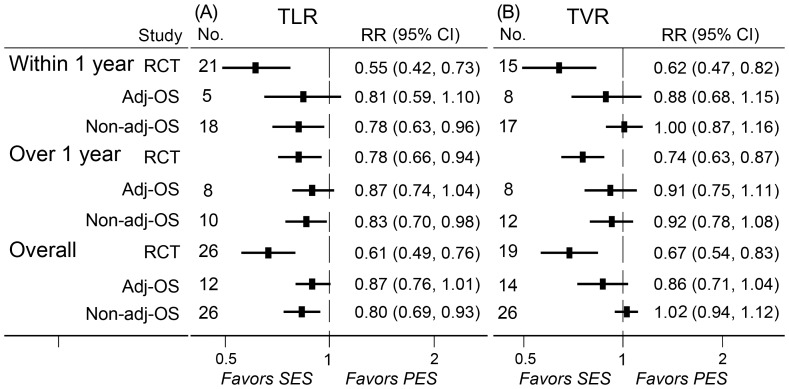
RRs and 95% CIs of TLR (A) and TVR (B) associated with SES versus PES. Short-term (≤1 year), long-term (>1 year), and overall-term TLR and TVR in patients treated with SES versus PES were evaluated. Adj-OS  =  adjusted observational study; CI  =  confidence interval; No.  =  number of the studies; Non-adj OS  =  non-adjusted observational study; PES  =  paclitaxel-eluting stents; RCT  =  randomized controlled trial; RR  =  relative risk; SES  =  sirolimus-eluting stent; TLR  =  target lesion revascularization; TVR  =  target vessel revascularization.

A total of 26 randomized controlled trials, 12 adjusted observational studies, and 26 non-adjusted observational studies contributed to the analysis of TLR. SES was associated with a significant reduction in TLR compared with PES at short-, long-, and overall-term follow-up in randomized controlled trials and non-adjusted observational studies, while no significant but a trend of reduction was detected in adjusted observational studies. Notably, the magnitudes of reducing TLR in randomized controlled trials were more evident than those in observational studies (**Fig. 2A**).

### Target vessel revascularization

A total of 19 randomized controlled trials, 14 adjusted observational studies, and 26 non-adjusted observational studies contributed to the analysis of TVR. A significant reduction of TVR was found in patients treated with SES at short-, long- and overall-term follow-up in randomized controlled trials (**Fig. 2B**). No significant difference in the rate of TVR was detected in observational studies.

### Major adverse cardiac event

**Figure 3:**
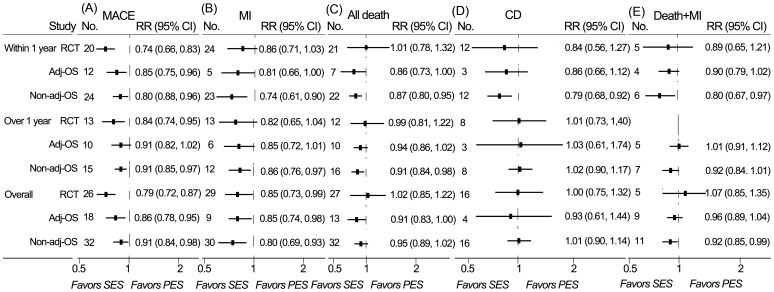
RRs and 95% CIs of other clinical endpoints associated with SES versus PES. MACE (A), MI (B), all-cause death (C), cardiac death (D), death/MI (E) were evaluated in Short-term (≤1 year), long-term (>1 year), and overall-term follow-up. Adj-OS  =  adjusted observational study; CD  =  cardiac death; CI  =  confidence interval; Death + MI  =  the composite of death and myocardial infarction; MACE  =  major adverse cardiac event; MI  =  myocardial infarction; No.  =  number of the studies; Non-adj OS  =  non-adjusted observational study; PES  =  paclitaxel-eluting stents; RCT  =  randomized controlled trial; RR  =  relative risk; SES  =  sirolimus-eluting stent.

A total of 26 randomized controlled trials, 18 adjusted observational studies, and 32 non-adjusted observational studies contributed to the analysis of MACE. SES was associated with a significant reduction of MACE compared with PES at short-, long-, and overall-term follow-up in randomized controlled trials, adjusted and non-adjusted observational studies, except that the difference of long-term rates of MACE did not reach a statistic level in adjusted observational studies (**Fig. 3A**).

### Myocardial infarction

A total of 29 randomized controlled trials, 9 adjusted observational studies, and 30 non-adjusted observational studies contributed to the analysis of myocardial infarction. SES was associated with significantly lower rates of myocardial infarction than PES at overall-term follow-up in randomized controlled trials, adjusted and non-adjusted observational studies (**Fig. 3B**). Significant reduction of MI with SES was also observed at short-term follow-up in adjusted observational studies, short and long-term follow-up in non-adjusted observational studies (**Fig. 3B**).

### All-cause death

A total of 27 randomized controlled trials, 13 adjusted observational studies, and 32 non-adjusted observational studies contributed to the analysis of all-cause death. No association of rates of all-cause death with SES was detected in randomized controlled trials, while a trend of association was found in adjusted observational studies. Meanwhile, SES-treated patients showed a significantly lower mortality compared to those receiving PES in adjusted observational studies at short and overall-term follow-up, and in non-adjusted observational studies at short and long-term follow-up (**Fig. 3C**).

### Cardiac death

A total of 16 randomized controlled trials, 4 adjusted observational studies, and 16 non-adjusted observational studies contributed to the analysis of cardiac death. SES was associated with significantly lower short-term rate of cardiac death in non-adjusted observational studies. There was no significant difference between SES and PES in cardiac death in randomized controlled trials and adjusted observational studies (**Fig. 3D**).

### Death or myocardial infarction

A total of 5 randomized controlled trials, 9 adjusted observational studies, and 11 non-adjusted observational studies contributed to the analysis of the composite of death and MI. SES reduced rates of death/MI in non-adjusted observational studies at short-term and overall follow-up. No significant association was detected in randomized controlled trials and adjusted observational studies (**Fig. 3E**).

### Stent thrombosis

**Figure 4:**
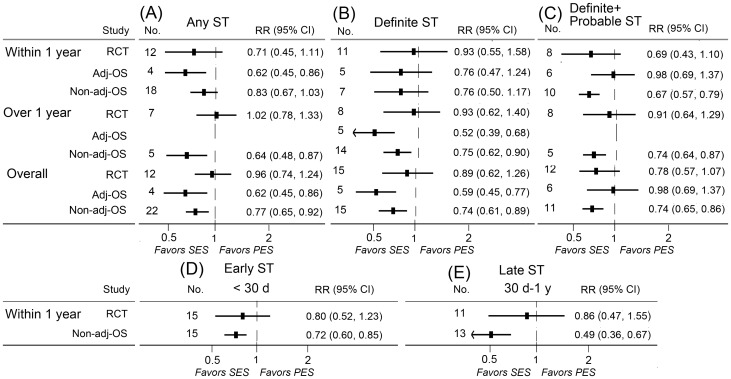
RRs and 95% CIs of stent thrombosis associated with SES versus PES. Short-term (≤1 year), long-term (>1 year), and overall-term stent thrombosis in patients treated with SES versus PES were evaluated. Short-term stent thrombosis was divided into early (≤30 days) and late (30 days-1 year) stages. Adj-OS  =  adjusted observational study; CI  =  confidence interval; No.  =  number of the studies; Non-adj OS  =  non-adjusted observational study; PES  =  paclitaxel-eluting stents; RCT  =  randomized controlled trial; RR  =  relative risk; SES  =  sirolimus-eluting stent; ST  =  stent thrombosis.

A total of 23 randomized controlled trials, 14 adjusted observational studies, and 31 non-adjusted observational studies contributed to the analysis of stent thrombosis. SES was associated with significant reductions of short-term rates of any stent thrombosis, long-term rates of definite stent thrombosis in adjusted observational studies. Significant reduced long-term rates of any stent thrombosis, definite stent thrombosis, definite or probable stent thrombosis, and short-term rates of definite or probable stent thrombosis (including early stent thrombosis and late thrombosis) were found in patients treated with SES in non-adjusted observational studies. No significant association of SES with stent thrombosis was found in randomized controlled trials (**Fig. 4**).

### Restenosis

**Figure 5:**
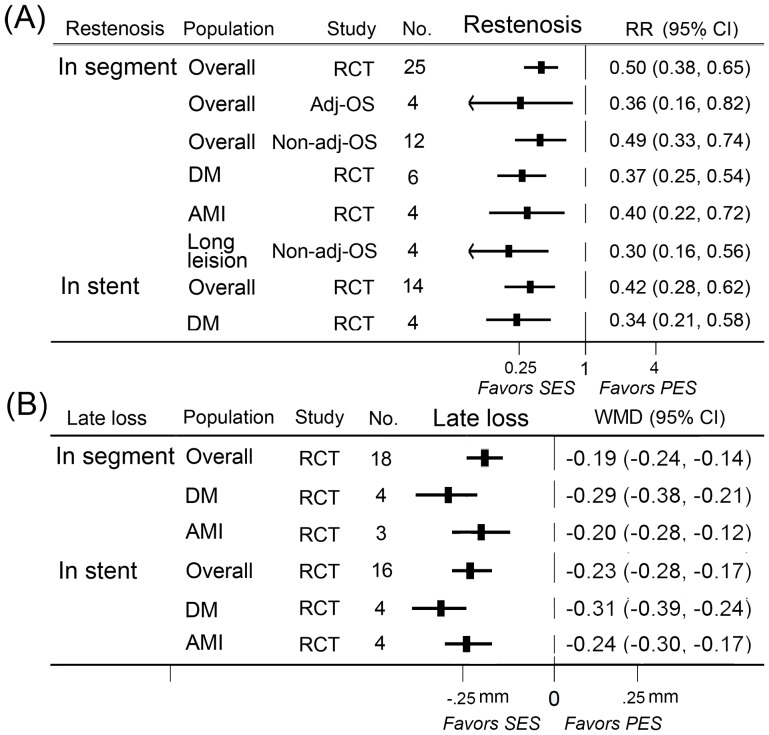
RRs/WMDs and 95% CIs of angiographic endpoints associated with SES versus PES. Angiographic outcomes were in-segment restenosis (A), in-stent restenosis (A), and in-segment late lumen loss (B), in-stent late lumen loss (B). Short-term (≤1 year), long-term (>1 year), and overall-term angiographic endpoints in patients treated with SES versus PES were evaluated. The overall population and subgroups of diabetes mellitus, acute myocardial infarction, and long lesion were included. Adj-OS  =  adjusted observational study; CI  =  confidence interval; No.  =  number of the studies; Non-adj OS  =  non-adjusted observational study; PES  =  paclitaxel-eluting stents; RCT  =  randomized controlled trial; RR  =  relative risk; SES  =  sirolimus-eluting stent; WMD  =  weighted mean difference.

A total of 25 randomized controlled trials, 4 adjusted observational studies, and 12 non-adjusted observational studies contributed to the analysis of in-segment or in-stent restenosis. SES was significantly more effective in reducing rates of in-segment restenosis and in-stent restenosis than PES in randomized controlled trials and adjusted or non-adjusted observational studies (**Fig. 5A**).

### Late lumen loss

18 randomized controlled trials contributed to the analysis of in-segment or in-stent late lumen loss. SES was associated with a significant reduction in in-segment and in-stent late lumen loss in randomized controlled trials (**Fig. 5B**).

With respect to most of the outcomes of interest, no evidence of publication bias was detected by performing the Begg's tests (**File S4**) and by visually inspecting the funnel plots (**File S5**). We evaluated the consistency of our findings by performing sensitivity analyses. The results were largely consistent in the sensitivity analyses, including in studies with low bias risk and in studies with 6 month's minimum clopidogrel use (**Table S2 and Table S3**), except that SES was more efficacious in reducing short-term rates of TVR in adjusted observational studies and rates of any stent thrombosis in non-adjusted observational studies with over 6 months of clopidogrel use. Sensitivity analyses that excluded all observational studies which reported ORs showed similar results.

### Meta-analyses in population with diabetes

**Figure 6:**
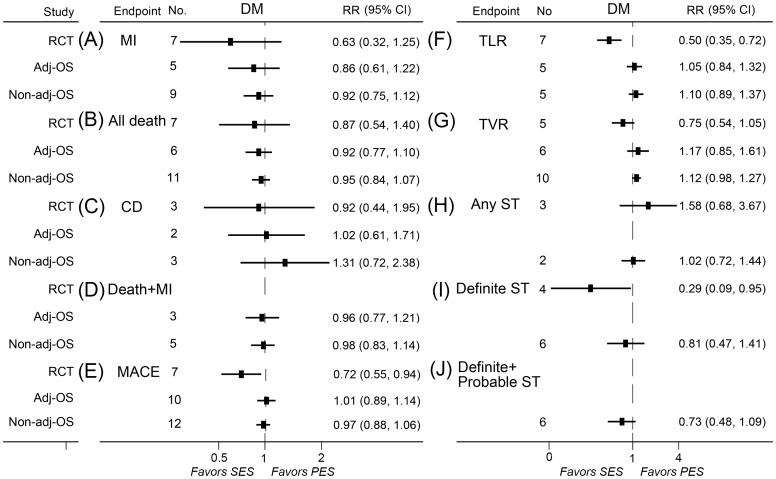
RRs and 95% CIs of clinical endpoints associated with SES versus PES in diabetes population. Adj-OS  =  adjusted observational study; CD  =  cardiac death; CI  =  confidence interval; Death + MI  =  the composite of death and myocardial infarction; DM  =  diabetes mellitus; MACE  =  major adverse cardiac event; MI  =  myocardial infarction; No.  =  number of the studies; Non-adj OS  =  non-adjusted observational study; PES  =  paclitaxel-eluting stents; RCT  =  randomized controlled trial; RR  =  relative risk; SES  =  sirolimus-eluting stent; ST  =  stent thrombosis; TLR  =  target lesion revascularization; TVR  =  target vessel revascularization.

A total of 10 randomized controlled trials, 12 adjusted observational studies, and 13 non-adjusted observational studies contributed to the analyses in diabetes population. SES significantly reduced the rates of MACE, TLR, definite stent thrombosis, in-segment restenosis, in-stent restenosis, in-segment LLL, and in-stent LLL in randomized controlled trials as compared with PES (**Fig. 5**, **Fig. 6**).

### Meta-analyses in population with acute myocardial infarction

**Figure 7:**
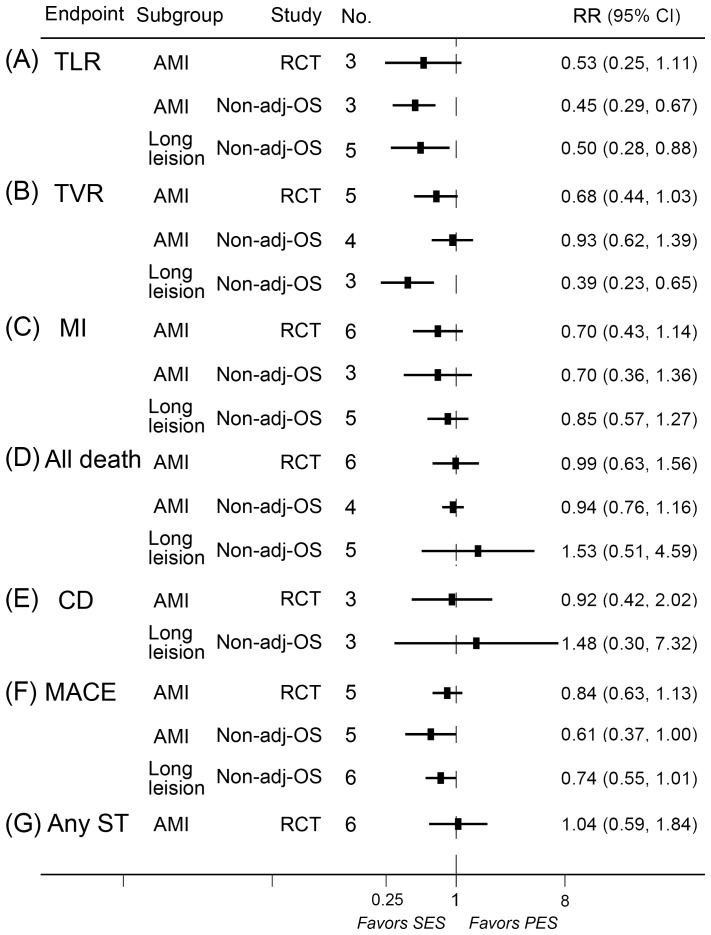
RRs and 95% CIs of clinical endpoints associated with SES versus PES in patient with AMI and long-lesions. Adj-OS  =  adjusted observational study; AMI  =  acute myocardial infarction; CD  =  cardiac death; CI  =  confidence interval; MACE  =  major adverse cardiac event; MI  =  myocardial infarction; No.  =  number of the studies; Non-adj OS  =  non-adjusted observational study; PES  =  paclitaxel-eluting stents; RCT  =  randomized controlled trial; RR  =  relative risk; SES  =  sirolimus-eluting stent; ST  =  stent thrombosis; TLR  =  target lesion revascularization; TVR  =  target vessel revascularization.

A total of 6 randomized controlled trials and 5 non-adjusted observational studies contributed in the analyses in population with acute myocardial infarction. SES significantly reduced the rates of MACE and TLR in non-adjusted observational studies and rates of in-segment restenosis, in-segment LLL, and in-stent LLL in randomized controlled trials compared with PES (**Fig. 5**, **Fig. 7**).

### Meta-analyses in population with long coronary lesions

A total of 7 studies contributed in the analyses in population with long coronary lesions. SES significantly reduced the rates of TLR, TVR, in-segment restenosis compared with PES (**Fig. 5**, **Fig. 7**).

## Discussion

In the present meta-analysis of head-to-head comparison of SES versus PES, we pooled together results from 76 studies including >15000 patients in randomized controlled trials and >70000 patients in adjusted observational studies. To the best of our knowledge, this is the largest study evaluating the relative efficiency and safety of SES with PES, summing up all the published and unpublished evidence. Furthermore, this is the first comprehensive attempt to make analyses of observational studies, which may represent real-world practice and often take in much more patients than randomized controlled trials. Analyses of these large-scale observational studies are more powered to detect differences in low-frequency safety events [21], being complementary to randomized controlled trials because of their small to modest sizes of population.

In our study, we found that SES-treated patients had a significantly lower rate of TLR. The reduction rates of TLR were 45%, 22%, and 39% at short-, long- and overall-term follow-up respectively in randomized controlled trials. Notably, a similar but less prominent trend was found in observational studies, with SES reducing TLR rates by 19%, 13%, and 13% at short-, long- and overall-term follow-up respectively in adjusted observational studies. Several prior analyses comparing DES with BMS showed similar patterns [4], [22]. Eric *et al* performed a meta-analysis of 8 randomized trials and 5 observational studies comparing DES with BMS in primary PCI at long-term follow-up, and demonstrated that patients receiving DES had a significant lower risk of TLR than BMS in randomized controlled trials (OR = 0.48, 95% CI = 0.37–0.61), but not significant in observational studies (OR = 0.52, 95% CI = 0.18–1.48) [22]. Several reasons may account for the discrepancies. High levels of mandated angiographic follow-up and absences of lesion complexity of randomized controlled trials might overestimate the efficacy of DES in these studies [21]. Similar explanations could be applied to our study. Notwithstanding the differences of magnitude in reducing TLR between randomized controlled trials and observational analyses, our study gave a clear conclusion that SES was more effective in reducing TLR. Largely similar results were obtained regarding TVR.

With respect to all-cause mortality, patients receiving SES were significantly lower than those receiving PES in observational studies, but not in randomized controlled trials. For instance, at short-term follow-up, SES reduced all-cause mortality by 14% in adjusted observational studies, but the benefit was not evident in randomized controlled trials (RR = 1.01, 95% CI = 0.78–1.32). In a comprehensive meta-analysis comparing safety and efficiency of DES with BMS, DES significantly reduces mortality by 22% in observational studies, but the effect is attenuated in RCTs (RR = 0.97, 95% CI = 0.81–1.15) [21]. The similar pattern observed in our study and others [23] which compare DES with BMS might not be fully interpreted with the differences of contents of these studies, but should be interpreted with the nature of study designs as well. Observational studies often enroll a large number of patients and thus are more powerful to detect differences in low-frequency events. In our study, 10000 patients were included in the analysis of all-cause mortality in randomized controlled trials, while over 50000 patients contributed to the analysis in observational studies. Nonetheless, absolute reliance should not be given to observational studies. Observational studies might overestimate treatment effects as compared with RCTs [24], because of its “as-treated” analysis (not intention-to-treat analysis in RCTs), publication bias and other bias such as residual confounding etc. A similar but more evident benefit was found in SES regarding myocardial infarction, which was not a surprise because it was consistent with the prior published meta-analysis by Bangalore and colleagues [8]. Similarly in this earlier work, there was a reduction in the risk of myocardial infarction with SES, at both short- and overall-term, compared with PES.

Although drug-eluting stents are more effective in reducing rate of restenosis than bare metal stents, concerns on their safety have also been voiced that DES may increase the risk of stent thrombosis [25]. In our study, the incidence of any stent thrombosis was 3.0%/3.2%, definite and probable stent thrombosis was 1.6%/2.1%, and definite stent thrombosis was 1.3%/1.5% for patients receiving SES and PES respectively in randomized controlled trials at overall-term follow-up. No significant difference in stent thrombosis was found between SES and PES in randomized controlled trials, which was consistent with most of the prior meta-analyses except those of Schomig *et al.* [6] and Bangalore *et al.* [8] However, in the study of Schomig *et al.*, stent thrombosis was not reported as what the Academic Research Consortium (ARC) criteria defined–definite, definite or probable stent thrombosis. Besides, data from this meta-analysis differed much from that of our study because a large part of the data were directly obtained from principal investigators or from abstracts in international meetings as many of the trials were not published yet then in peer-reviewed journals. Bangalore *et al.* performed a mixed treatment comparison meta-analysis (MTC meta-analysis), also known as network meta-analysis, to compare the efficacy and safety outcomes in BMS and DES. “MTC meta-analysis is an extension that allows the combination of direct with indirect comparisons”, which, according to the Cochrane Collaboration's guidance, are not randomized, but are “observational findings across trials, and may suffer the biases of observational studies, for example due to confounding”, even if they address high-quality randomized controlled trials [10], [26]. Similar to the work of Bangalore and colleagues, in our observational studies (adjusted and non-adjusted), risks of any stent thrombosis, definite stent thrombosis, and definite or probable stent thrombosis were lower in SES-treated patients than PES at long- and overall-term follow-up, except that no difference was found at overall-term follow-up of adjusted observational studies. In addition, SES was superior over PES in reducing both early (<30 days) and late (30 days – 1 year) stent thrombosis in non-adjusted observational studies.

Several limitations should be acknowledged in our study. First, both of these stents are not actively used in current clinical practice. Second, our meta-analysis were fully based on the published data, we did not contact the authors for any individual data. Third, not all the studies reported each of the outcomes of interest in our study, therefore selective reporting bias could not be excluded. Fourth, the definition of major adverse cardiac events—an important endpoint in our study—was not completely consistent across the studies, which though, is unlikely to have a relevant impact on the results of our meta-analysis. Other outcomes of interest were also based on reported definitions of the studies included. Fifth, in the analyses of patients with long coronary lesions, data from randomized controlled trials and non-adjusted observational studies were pooled together due to the limited number of studies involved.

## Conclusions

In this large head-to-head comparative meta-analysis of 76 studies including more than 15000 patients in randomized controlled trials and over 70000 patients in adjusted observational studies, SES significantly reduced the short-, long- and overall-term risk of TLR/TVR, MACE and restenosis, and overall-term risk of myocardial infarction in randomized controlled trials as compared with PES. Lower rates of death and stent thrombosis were also observed in observational studies in SES-treated patients than PES.

## Supporting Information

File S1 PRISMA checklist summary for the meta-analysis.
(DOC)

File S2 Bibliography of the 95 articles involving 76 studies included in the meta-analysis.
(DOC)

File S3 The quality scales of studies included in the meta-analysis.
(XLSX)

File S4 P values for heterogeneity and publication bias (Begg's test) in the meta-analyses.
(XLSX)

File S5 Funnel plots of the meta-analyses to detect publication bias.
(DOC)

Table S1 Characteristics of studies included in the meta-analysis.
(DOC)

Table S2 Sensitivity analyses of outcomes in different subgroups.
(DOC)

Table S3 Sensitivity analyses of angiographic outcomes of different subgroups in randomized controlled trials.
(DOC)
