# Supplementary material for: Head-to-Head Comparison of Sirolimus-Eluting Stents versus Paclitaxel-Eluting Stents in Patients Undergoing Percutaneous Coronary Intervention: A Meta-Analysis of 76 Studies
Source: PLoS One. 2014 May 20;9(5):e97934. doi: 10.1371/journal.pone.0097934 (PMC4028235; doi:10.1371/journal.pone.0097934)
Supplement: File S2 — Bibliography of the 95 articles involving 76 studies included in the meta-analysis. (DOC) [file pone.0097934.s002.doc]

**ELIGIBLE STUDIES AND REFERENCES**

Of the 2967 potentially relevant articles initially screened, 95 articles involving 76 studies met our inclusion criteria and were included in the meta-analysis. Among them, 33 studies (7590 persons in SES arm, 7520 in PES) in 39 articles were randomized controlled trials, 27 studies (39904 persons in SES arm, 31694 in PES) in 37 articles were adjusted observational studies, and 41 studies (44734 persons in SES arm, 33240 in PES) in 51 articles were non-adjusted observational studies. 32 articles describing 25 studies reported both adjusted and non-adjusted results of outcomes. The recommended duration of clopidogrel therapy was at least 6 months in most studies, while 2 randomized controlled trials and 9 observational studies failed to report the length of dual antiplatelet therapy.

1. Di Lorenzo E, Sauro R, Varricchio A, Capasso M, Lanzillo T, et al. (2009) Benefits of drug-eluting stents as compared to bare metal stent in ST-segment elevation myocardial infarction: Four year results of the PaclitAxel or Sirolimus-Eluting stent vs bare metal stent in primary angiOplasty (PASEO) randomized trial. Am Heart J 158: e43-e50.

2. Kim BS, You SH, Hong SJ, Kim MH, Cha KS, et al. (2009) Comparison of 3-year clinical outcomes between sirolimus-versus paclitaxel-eluting stents in diabetic patients: Prospective randomized multicenter trial. Am J Cardiol 104: 158D-159D.

3. Galloe AM, Thuesen L, Kelbaek H, Thayssen P, Rasmussen K, et al. (2008) Comparison of paclitaxel- and sirolimus-eluting stents in everyday clinical practice: the SORT OUT II randomized trial. JAMA 299: 409-416.

4. Han YL, Wang XZ, Jing QM, Wang SL, Ma YY, et al. (2006) Comparison of Rapamycin and Paclitaxel eluting stent in patients with multi-vessel coronary disease. Zhonghua Xin Xue Guan Bing Za Zhi 34: 123-126.

5. Juwana YB, Suryapranata H, Ottervanger JP, De Luca G, van't Hof AW, et al. (2009) Comparison of rapamycin- and paclitaxel-eluting stents in patients undergoing primary percutaneous coronary intervention for ST-elevation myocardial infarction. Am J Cardiol 104: 205-209.

6. Fukumoto A, Otsuji S, Takiuchi S, Ikushima M, Asano K, et al. (2011) Comparison of real-world clinical outcomes between Cypher- and Taxus-eluting stents: The GARA-GARA study. Cardiovascular Intervention and Therapeutics 26: 202-208.

7. Lee CW, Park DW, Lee SH, Kim YH, Hong MK, et al. (2009) Comparison of the efficacy and safety of zotarolimus-, sirolimus-, and paclitaxel-eluting stents in patients with ST-elevation myocardial infarction. Am J Cardiol 104: 1370-1376.

8. Maeng M, Jensen LO, Galloe AM, Thayssen P, Christiansen EH, et al. (2009) Comparison of the sirolimus-eluting versus paclitaxel-eluting coronary stent in patients with diabetes mellitus: the diabetes and drug-eluting stent (DiabeDES) randomized angiography trial. Am J Cardiol 103: 345-349.

9. Hong SJ, Kim MH, Cha KS, Park HS, Chae SC, et al. (2010) Comparison of three-year clinical outcomes between sirolimus-versus paclitaxel-eluting stents in diabetic patients: prospective randomized multicenter trial. Catheter Cardiovasc Interv 76: 924-933.

10. Kang WC, Ahn T, Lee K, Han SH, Shin EK, et al. (2011) Comparison of zotarolimus-eluting stents versus sirolimus-eluting stents versus paclitaxel-eluting stents for primary percutaneous coronary intervention in patients with ST-elevation myocardial infarction: results from the Korean Multicentre Endeavor (KOMER) acute myocardial infarction (AMI) trial. EuroIntervention 7: 936-943.

11. Park DW, Kim YH, Yun SC, Kang SJ, Lee SW, et al. (2010) Comparison of zotarolimus-eluting stents with sirolimus- and paclitaxel-eluting stents for coronary revascularization: the ZEST (comparison of the efficacy and safety of zotarolimus-eluting stent with sirolimus-eluting and paclitaxel-eluting stent for coronary lesions) randomized trial. J Am Coll Cardiol 56: 1187-1195.

12. Kim BK, Ko YG, Oh S, Kim JS, Kang WC, et al. (2010) Comparisons of the effects of stent eccentricity on the neointimal hyperplasia between sirolimus-eluting stent versus paclitaxel-eluting stent. Yonsei Med J 51: 823-831.

13. de Lezo J, Medina A, Pan M, Romero M, Delgado A, et al. (2005) Drug-eluting stents for complex lesions: randomized rapamycin versus paclitaxel CORPAL study (abstr). J Am Coll Cardiol 45.

14. Pan M, Suarez de Lezo J, Medina A, Romero M, Delgado A, et al. (2007) Drug-eluting stents for the treatment of bifurcation lesions: a randomized comparison between paclitaxel and sirolimus stents. Am Heart J 153: 15 e11-17.

15. Kim MH, Hong SJ, Cha KS, Park HS, Chae SC, et al. (2008) Effect of Paclitaxel-eluting versus sirolimus-eluting stents on coronary restenosis in Korean diabetic patients. J Interv Cardiol 21: 225-231.

16. Raber L, Wohlwend L, Wigger M, Togni M, Wandel S, et al. (2011) Five-year clinical and angiographic outcomes of a randomized comparison of sirolimus-eluting and paclitaxel-eluting stents: results of the Sirolimus-Eluting Versus Paclitaxel-Eluting Stents for Coronary Revascularization LATE trial. Circulation 123: 2819-2828, 2816 p following 2828.

17. Cervinka P, Costa MA, Angiolillo DJ, Spacek R, Bystron M, et al. (2006) Head-to-head comparison between sirolimus-eluting and paclitaxel-eluting stents in patients with complex coronary artery disease: an intravascular ultrasound study. Catheter Cardiovasc Interv 67: 846-851.

18. Tomai F, Reimers B, De Luca L, Galassi AR, Gaspardone A, et al. (2008) Head-to-head comparison of sirolimus- and paclitaxel-eluting stent in the same diabetic patient with multiple coronary artery lesions: a prospective, randomized, multicenter study. Diabetes care 31: 15-19.

19. Kang KW, Ko YG, Shin DH, Kim JS, Kim BK, et al. (2012) Impact of positive peri-stent vascular remodeling after sirolimus-eluting and paclitaxel-eluting stent implantation on 5-year clinical outcomes: intravascular ultrasound analysis from the Poststent Optimal Stent Expansion Trial multicenter randomized trial. Circ J 76: 1102-1108.

20. Kaiser C, Brunner-La Rocca HP, Buser PT, Bonetti PO, Osswald S, et al. (2005) Incremental cost-effectiveness of drug-eluting stents compared with a third-generation bare-metal stent in a real-world setting: randomised Basel Stent Kosten Effektivitäts Trial (BASKET). Lancet 366: 921-929.

21. Kim HS, Lee JH, Lee SW, Kim YH, Park JH, et al. (2011) Long-term safety and efficacy of sirolimus- vs. paclitaxel-eluting stent implantation for acute ST-elevation myocardial infarction: 3-year follow-up of the PROSIT trial. Int J Cardiol 147: 253-257.

22. Briguori C, Airoldi F, Visconti G, Focaccio A, Caiazzo G, et al. (2011) Novel approaches for preventing or limiting events in diabetic patients (Naples-diabetes) trial: a randomized comparison of 3 drug-eluting stents in diabetic patients. Circ Cardiovasc Interv 4: 121-129.

23. Zhang Q, Zhang RY, Zhang JS, Hu J, Yang ZK, et al. (2006) One-year clinical outcomes of Chinese sirolimus-eluting stent in the treatment of unselected patients with coronary artery disease. Chin Med J 119: 165-168.

24. Guagliumi G, Musumeci G, Sirbu V, Bezerra HG, Suzuki N, et al. (2010) Optical coherence tomography assessment of in vivo vascular response after implantation of overlapping bare-metal and drug-eluting stents. JACC Cardiovasc Interv 3: 531-539.

25. Mehilli J, Kastrati A, Byrne RA, Bruskina O, Iijima R, et al. (2009) Paclitaxel- versus sirolimus-eluting stents for unprotected left main coronary artery disease. J Am Coll Cardiol 53: 1760-1768.

26. Dibra A, Kastrati A, Mehilli J, Pache J, Schuhlen H, et al. (2005) Paclitaxel-eluting or sirolimus-eluting stents to prevent restenosis in diabetic patients. N Engl J Med 353: 663-670.

27. Di Lorenzo E, De Luca G, Sauro R, Varricchio A, Capasso M, et al. (2009) The PASEO (PaclitAxel or Sirolimus-Eluting Stent Versus Bare Metal Stent in Primary Angioplasty) Randomized Trial. JACC Cardiovasc Interv 2: 515-523.

28. Goy JJ, Stauffer JC, Siegenthaler M, Benoit A, Seydoux C (2005) A prospective randomized comparison between paclitaxel and sirolimus stents in the real world of interventional cardiology: the TAXi trial. J Am Coll Cardiol 45: 308-311.

29. Lee JH, Kim HS, Lee SW, Park JH, Choi SW, et al. (2008) Prospective randomized comparison of sirolimus- versus paclitaxel-eluting stents for the treatment of acute ST-elevation myocardial infarction: pROSIT trial. Catheter Cardiovasc Interv 72: 25-32.

30. Petronio AS, De Carlo M, Branchitta G, Papini B, Ciabatti N, et al. (2007) Randomized comparison of sirolimus and paclitaxel drug-eluting stents for long lesions in the left anterior descending artery: an intravascular ultrasound study. J Am Coll Cardiol 49: 539-546.

31. Lee SW, Park SW, Kim YH, Yun SC, Park DW, et al. (2008) A randomized comparison of sirolimus- versus Paclitaxel-eluting stent implantation in patients with diabetes mellitus. J Am Coll Cardiol 52: 727-733.

32. Lee SW, Park SW, Kim YH, Yun SC, Park DW, et al. (2011) A randomized comparison of sirolimus- versus paclitaxel-eluting stent implantation in patients with diabetes mellitus: 4-year clinical outcomes of DES-DIABETES (drug-eluting stent in patients with DIABETES mellitus) trial. JACC Cardiovasc Interv 4: 310-316.

33. Mehilli J, Dibra A, Kastrati A, Pache J, Dirschinger J, et al. (2006) Randomized trial of paclitaxel- and sirolimus-eluting stents in small coronary vessels. Eur Heart J 27: 260-266.

34. Mehilli J, Byrne RA, Tiroch K, Pinieck S, Schulz S, et al. (2010) Randomized trial of paclitaxel- versus sirolimus-eluting stents for treatment of coronary restenosis in sirolimus-eluting stents: the ISAR-DESIRE 2 (Intracoronary Stenting and Angiographic Results: Drug Eluting Stents for In-Stent Restenosis 2) study. J Am Coll Cardiol 55: 2710-2716.

35. Morice MC, Colombo A, Meier B, Serruys P, Tamburino C, et al. (2006) Sirolimus- vs paclitaxel-eluting stents in de novo coronary artery lesions: the REALITY trial: a randomized controlled trial. JAMA 295: 895-904.

36. Kastrati A, Mehilli J, von Beckerath N, Dibra A, Hausleiter J, et al. (2005) Sirolimus-eluting stent or paclitaxel-eluting stent vs balloon angioplasty for prevention of recurrences in patients with coronary in-stent restenosis: a randomized controlled trial. JAMA 293: 165-171.

37. Kim YH, Park SW, Lee SW, Park DW, Yun SC, et al. (2006) Sirolimus-eluting stent versus paclitaxel-eluting stent for patients with long coronary artery disease. Circulation 114: 2148-2153.

38. Berger A, Stauffer JC, Seydoux C, Siegenthaler M, Benoit A, et al. (2007) Three-year follow-up of the first prospective randomized comparison between paclitaxel and sirolimus stents: the TAXi-LATE trial. Catheter Cardiovasc Interv 70: 163-166.

39. Billinger M, Beutler J, Taghetchian KR, Remondino A, Wenaweser P, et al. (2008) Two-year clinical outcome after implantation of sirolimus-eluting and paclitaxel-eluting stents in diabetic patients. Eur Heart J 29: 718-725.

40. Kaltoft A, Jensen LO, Maeng M, Tilsted HH, Thayssen P, et al. (2009) 2-year clinical outcomes after implantation of sirolimus-eluting, paclitaxel-eluting, and bare-metal coronary stents: results from the WDHR (Western Denmark Heart Registry). J Am Coll Cardiol 53: 658-664.

41. Bonello L, Buch AN, De Labriolle A, Roy P, Steinberg DH, et al. (2010) Clinical outcomes after implantation of small diameter (= 2.5 mm) sirolimus- versus paclitaxel-eluting stents. Int J Cardiol 144: 105-107.

42. Saia F, Piovaccari G, Manari A, Santarelli A, Benassi A, et al. (2006) Clinical outcomes for sirolimus-eluting stents and polymer-coated paclitaxel-eluting stents in daily practice: results from a large multicenter registry. J Am Coll Cardiol 48: 1312-1318.

43. Lee SR, Jeong MH, Ahn YK, Chae SC, Hur SH, et al. (2008) Clinical safety of drug-eluting stents in the Korea acute myocardial infarction registry. Circ J 72: 392-398.

44. Cosgrave J, Melzi G, Corbett S, Biondi-Zoccai GG, Agostoni P, et al. (2007) Comparable clinical outcomes with paclitaxel- and sirolimus-eluting stents in unrestricted contemporary practice. J Am Coll Cardiol 49: 2320-2328.

45. Simonton CA, Brodie B, Cheek B, Krainin F, Metzger C, et al. (2007) Comparative Clinical Outcomes of Paclitaxel- and Sirolimus-Eluting Stents. Results From a Large Prospective Multicenter Registry-STENT Group. J Am Coll Cardiol 50: 1214-1222.

46. Balducelli M, Ortolani P, Marzaroli P, Piovaccari G, Menozzi A, et al. (2010) Comparison of 2-year clinical outcomes with sirolimus and paclitaxel-eluting stents for patients with diabetes: results of the Registro Regionale AngiopLastiche Emilia-Romagna Registry. Catheter Cardiovasc Interv 75: 327-334.

47. Park K, Park KW, Rha SW, Bae JH, Hur SH, et al. (2012) Comparison of 5-year clinical outcomes between sirolimus-versus paclitaxel-eluting stent: Korean multicenter network analysis of 9000-patient cohort. Circ Cardiovasc Interv 5: 174-184.

48. Hachinohe D, Jeong MH, Saito S, Kim MC, Cho KH, et al. (2012) Comparison of drug-eluting stents in acute myocardial infarction patients with chronic kidney disease. Korean Journal of Internal Medicine 27: 397-406.

49. Hannan EL, Racz M, Holmes DR, Sharma S, Katz S, et al. (2007) A comparison of mortality, myocardial infarction, and repeated revascularization for sirolimus-eluting and paclitaxel-eluting coronary stents. Am Heart J 154: 545-553.

50. Kim JS, Lee BH, Ko YG, Choi D, Jang Y, et al. (2008) Comparison of sirolimus-eluting stent and paclitaxel-eluting stent for long-term cardiac adverse events in diabetic patients: the Korean Multicenter Angioplasty Team (KOMATE) Registry. Catheter Cardiovasc Interv 72: 601-607.

51. Kim YH, Park SW, Lee CW, Hong MK, Gwon HC, et al. (2006) Comparison of sirolimus-eluting stent, paclitaxel-eluting stent, and bare metal stent in the treatment of long coronary lesions. Catheter Cardiovasc Interv 67: 181-187.

52. Daemen J, Tanimoto S, Garcia-Garcia HM, Kukreja N, van de Sande M, et al. (2007) Comparison of three-year clinical outcome of sirolimus- and paclitaxel-eluting stents versus bare metal stents in patients with ST-segment elevation myocardial infarction (from the RESEARCH and T-SEARCH Registries). Am J Cardiol 99: 1027-1032.

53. Daemen J, Wenaweser P, Tsuchida K, Abrecht L, Vaina S, et al. (2007) Early and late coronary stent thrombosis of sirolimus-eluting and paclitaxel-eluting stents in routine clinical practice: data from a large two-institutional cohort study. Lancet 369: 667-678.

54. Ko YG, Kim JS, Choi D, Hong MK, Min PK, et al. (2011) Five-year outcomes of sirolimus-eluting versus paclitaxel-eluting stents: a propensity matched study: clinical evidence of late catch-up? Int J Cardiol 152: 302-306.

55. Wenaweser P, Daemen J, Zwahlen M, van Domburg R, Juni P, et al. (2008) Incidence and correlates of drug-eluting stent thrombosis in routine clinical practice. 4-year results from a large 2-institutional cohort study. J Am Coll Cardiol 52: 1134-1140.

56. Iakovou I, Schmidt T, Bonizzoni E, Ge L, Sangiorgi GM, et al. (2005) Incidence, predictors and outcome of thrombosis after succesful implantation of drug-eluting stents. JAMA 293: 2126-2130.

57. Novack V, Cutlip D, Kleiman N, Pencina M, Mauri L, et al. (2009) In-Hospital and 1-Year Outcomes Among Unselected Percutaneous Coronary Intervention Patients Treated With Either Sirolimus- or Paclitaxel-Eluting Stents. Results From the EVENT (Evaluation of Drug Eluting Stents and Ischemic Events) Registry. JACC Cardiovasc Interv 2: 767-775.

58. Syed A, Collins S, Ben-Dor I, Li Y, Maluenda G, et al. (2009) Is there a difference in outcomes in patients with chronic renal insufficiency who are implanted with a sirolimus-eluting stent versus a paclitaxel-eluting stent? Am J Cardiol 104: 201D-202D.

59. Chieffo A, Park SJ, Meliga E, Sheiban I, Lee MS, et al. (2008) Late and very late stent thrombosis following drug-eluting stent implantation in unprotected left main coronary artery: a multicentre registry. Eur Heart J 29: 2108-2115.

60. Waksman R, Buch AN, Torguson R, Xue Z, Smith K, et al. (2007) Long-Term Clinical Outcomes and Thrombosis Rates of Sirolimus-Eluting Versus Paclitaxel-Eluting Stents in an Unselected Population With Coronary Artery Disease (REWARDS Registry). Am J Cardiol 100: 45-51.

61. Lee JY, Park DW, Yun SC, Lee SW, Kim YH, et al. (2009) Long-term clinical outcomes of sirolimus- versus paclitaxel-eluting stents for patients with unprotected left main coronary artery disease: analysis of the MAIN-COMPARE (revascularization for unprotected left main coronary artery stenosis: comparison of percutaneous coronary angioplasty versus surgical revascularization) registry. J Am Coll Cardiol 54: 853-859.

62. Ferenc M, Gick M, Kienzle RP, Bestehorn HP, Werner KD, et al. (2010) Long-term outcome of percutaneous catheter intervention for de novo coronary bifurcation lesions with drug-eluting stents or bare-metal stents. Am Heart J 159: 454-461.

63. Choi CU, Rha SW, Chen KY, Jin Z, Minami Y, et al. (2009) Mid-term angiographic benefit of sirolimus-eluting stents compared with paclitaxel-eluting stents in patients with acute myocardial infarction. Journal of Cardiology 54: 80-85.

64. Mayor M, Malik AZ, Minor Jr RJ, Deshpande MC, Strauss WE, et al. (2009) One-Year Outcomes from the TAXUS Express Stent Versus Cypher Stent. Am J Cardiol 103: 930-936.

65. Akin I, Bufe A, Eckardt L, Reinecke H, Richardt G, et al. (2010) Outcomes after differential use of drug-eluting stents in diabetic patients: 1-Year results from the DES.DE (Drug-Eluting Stent.DEutschland) registry. Catheter Cardiovasc Interv 76: 50-57.

66. Williams DO, Abbott JD, Kip KE (2006) Outcomes of 6906 patients undergoing percutaneous coronary intervention in the era of drug-eluting stents: report of the DEScover Registry. Circulation 114: 2154-2162.

67. Jensen LO, Tilsted HH, Thayssen P, Kaltoft A, Maeng M, et al. (2010) Paclitaxel and sirolimus eluting stents versus bare metal stents: long-term risk of stent thrombosis and other outcomes. From the Western Denmark Heart Registry. EuroIntervention 5: 898-905.

68. Cho Y, Yang HM, Park KW, Chung WY, Choi DJ, et al. (2010) Paclitaxel- versus sirolimus-eluting stents for treatment of ST-segment elevation myocardial infarction: with analyses for diabetic and nondiabetic subpopulation. JACC Cardiovasc Interv 3: 498-506.

69. Ishikawa T, Nakano Y, Mutoh M (2012) Retrospective comparison of midterm clinical and angiographic outcomes after the implantation of paclitaxel- and sirolimus-eluting stents for de novo coronary complex lesions in nonrandomized Japanese patients. Intern Med 51: 2695-2701.

70. Millauer N, Juni P, Hofmann A, Wandel S, Bhambhani A, et al. (2011) Sirolimus versus paclitaxel coronary stents in clinical practice. Catheter Cardiovasc Interv 77: 5-12.

71. Song YB, Hahn JY, Choi SH, Choi JH, Lee SH, et al. (2010) Sirolimus- versus paclitaxel-eluting stents for the treatment of coronary bifurcations results: from the COBIS (Coronary Bifurcation Stenting) Registry. J Am Coll Cardiol 55: 1743-1750.

72. Suh JW, Park JS, Cho HJ, Kim MS, Kang HJ, et al. (2007) Sirolimus-eluting stent showed better one-year outcomes than paclitaxel-eluting stent in a real life setting of coronary intervention in Koreans. Int J Cardiol 117: 31-36.

73. Hanna NN, Gaglia MA, Jr., Torguson R, Ben-Dor I, Gonzalez MA, et al. (2010) Three-year outcomes following sirolimus- versus paclitaxel-eluting stent implantation in an unselected population with coronary artery disease (from the REWARDS Registry). Am J Cardiol 106: 504-510.

74. Hoye A, van Mieghem CA, Ong AT, Aoki J, Rodriguez Granillo GA, et al. (2005) Treatment of de novo bifurcation lesions: comparison of Sirolimus- and Paclitaxel-eluting stents. EuroIntervention 1: 24-30.

75. Ong ATL, Serruys PW, Aoki J, Hoye A, Van Mieghem CAG, et al. (2005) The unrestricted use of paclitaxel- versus sirolimus-eluting stents for coronary artery disease in an unselected population: One-year results of the Taxus-Stent Evaluated at Rotterdam Cardiology Hospital (T-SEARCH) registry. J Am Coll Cardiol 45: 1135-1141.

76. Simsek C, Magro M, Boersma E, Onuma Y, Nauta ST, et al. (2010) The unrestricted use of sirolimus- and paclitaxel-eluting stents results in better clinical outcomes during 6-year follow-up than bare-metal stents: An analysis of the RESEARCH (Rapamycin-Eluting Stent Evaluated at Rotterdam Cardiology Hospital) and T-SEARCH (TaxusStent Evaluated at Rotterdam Cardiology Hospital) registries. JACC Cardiovasc Interv 3: 1051-1058.

77. Kim U, Kim DK, Seol SH, Yang TH, Kim DI, et al. (2010) Clinical and angiographic outcomes of drug-eluting stents in patients with large vessel and single coronary artery lesion. Clin Cardiol 33: 340-344.

78. Qiao SB, Hou Q, Xu B, Yang YJ, Chen JL, et al. (2006) The clinical efficacy of sirolimus-eluting stents versus paclitaxel-eluting stents in complex and diffuse coronary lesions. Zhonghua nei ke za zhi 45: 985-987.

79. Kim KH, Koo BK, Min HS, Park SK, Kim CH, et al. (2012) Comparison of drug-eluting versus bare-metal stent implantation in ST-elevation myocardial infarction patients with renal insufficiency: results from the national registry in Korea. Int J Cardiol 154: 71-77.

80. Yang YJ, Xu B, Kang S, Pei WD, Chen JL, et al. (2007) Comparison of in-hospital and long-term outcomes between a Cypher stent and a Taxus stent in Chinese diabetic patients with coronary artery disease. Chin Med J 120: 1868-1873.

81. Hur SH, Cho YK, Nam CW, Kim H, Han SW, et al. (2009) Comparison of long-term outcomes following sirolimus-eluting stent vs paclitaxel-eluting stent implantation in patients with long calcified coronary lesions. Clin Cardiol 32: 633-638.

82. Lee MG, Jeong MH, Ahn Y, Cho JG, Park JC, et al. (2011) Comparison of paclitaxel-, sirolimus-, and zotarolimus-eluting stents in patients with acute ST-segment elevation myocardial infarction and metabolic syndrome. Circ J 75: 2120-2127.

83. Chen S, Zhang J, Ye F, Zhu Z, Lin S, et al. (2008) Crush stenting with paclitaxel-eluting or sirolimus-eluting stents for the treatment of coronary bifurcation lesions. Angiology 59: 475-483.

84. Kataoka Y, Yagi N, Kokubu N, Kasahara Y, Abe M, et al. (2011) Efficacy of paclitaxel-eluting stent in patients with impaired glucose tolerance--comparison with sirolimus-eluting stent. Circ J 75: 868-873.

85. Cicek D, Pekdemir H, Kalay N, Binici S, Altay H, et al. (2010) Efficacy of sirolimus-eluting stents compared with paclitaxel-eluting stents in an unselected population with coronary artery disease: 24-month outcomes of patients in a prospective non-randomized registry in southern Turkey. International Journal of Medical Sciences 7: 191-196.

86. Chong E, Poh KK, Liang S, Hou XM, Tan HC (2010) Eighteen-month clinical safety and efficacy outcomes of sirolimus-, paclitaxel- and zotarolimus-drug eluting stents in diabetic patients undergoing percutaneous coronary intervention for complex coronary artery stenosis. Ann Acad Med Singapore 39: 381-384.

87. Stankovic G, Cosgrave J, Chieffo A, Iakovou I, Sangiorgi G, et al. (2006) Impact of sirolimus-eluting and Paclitaxel-eluting stents on outcome in patients with diabetes mellitus and stenting in more than one coronary artery. Am J Cardiol 98: 362-366.

88. Park GM, Park DW, Kim YG, Cho SW, Sun BJ, et al. (2013) Long-term luminal change after drug-eluting stent implantation: Serial angiographic follow-up study of the ZEST randomized trial. Catheter Cardiovasc Interv 81: 274-282.

89. Lee MS, Tarantini G, Xhaxho J, Yang T, Ehdaie A, et al. (2010) Sirolimus- versus paclitaxel-eluting stents for the treatment of cardiac allograft vasculopathy. JACC Cardiovasc Interv 3: 378-382.

90. Latib A, Cosgrave J, Godino C, Qasim A, Corbett SJ, et al. (2008) Sirolimus-eluting and paclitaxel-eluting stents for the treatment of coronary bifurcations. Am Heart J 156: 745-750.

91. Katritsis DG, Korovesis S, Karabinos I, Giazitzoglou E, Theodorou S, et al. (2006) Sirolimus-versus paclitaxel-eluting stents: a comparison of two consecutive series in routine clinical practice. J Interv Cardiol 19: 31-37.

92. Trabattoni D, Fabbiocchi F, Montorsi P, Ravagnani P, Galli S, et al. (2007) Stent thrombosis after sirolimus- and paclitaxel-eluting stent implantation in daily clinical practice: Analysis of a single center registry. Catheter Cardiovasc Interv 70: 415-421.

93. Ong AT, Hoye A, Aoki J, van Mieghem CA, Rodriguez Granillo GA, et al. (2005) Thirty-day incidence and six-month clinical outcome of thrombotic stent occlusion after bare-metal, sirolimus, or paclitaxel stent implantation. J Am Coll Cardiol 45: 947-953.

94. Erdim R, Helvacioglu F, Gormez S, Karabay KO, Aytekin V (2012) Two-year follow-up of sirolimus-eluting stents versus paclitaxel-eluting stents in acute myocardial infarction. International Journal of Angiology 21: 53-57.

95. Raber L, Magro M, Stefanini GG, Kalesan B, van Domburg RT, et al. (2012) Very late coronary stent thrombosis of a newer-generation everolimus-eluting stent compared with early-generation drug-eluting stents: a prospective cohort study. Circulation 125: 1110-1121.
