# Supplementary material for: Head-to-Head Comparison of Sirolimus-Eluting Stents versus Paclitaxel-Eluting Stents in Patients Undergoing Percutaneous Coronary Intervention: A Meta-Analysis of 76 Studies
Source: PLoS One. 2014 May 20;9(5):e97934. doi: 10.1371/journal.pone.0097934 (PMC4028235; doi:10.1371/journal.pone.0097934)

**FUNNEL PLOTS**

**RCTs WITHIN 1 year**

TLR TVR


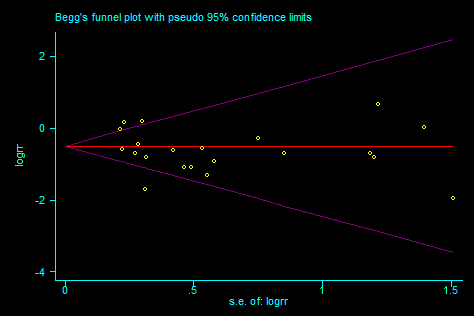

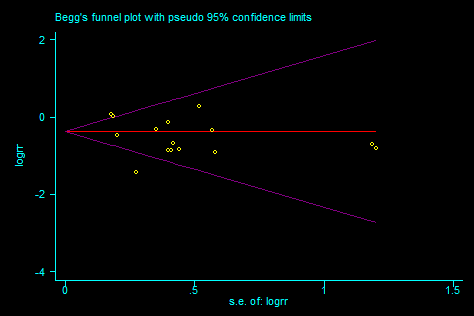


MACE Myocardial infarction


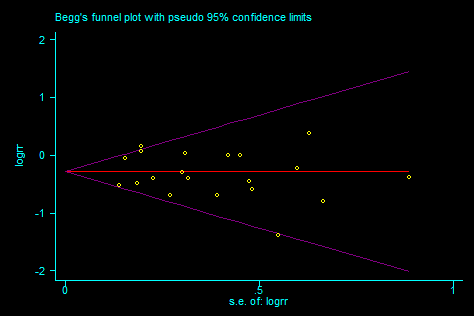

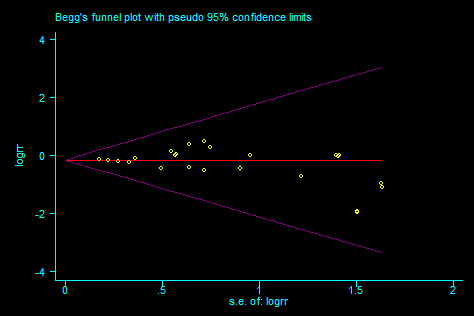


All-cause death Cardiac death


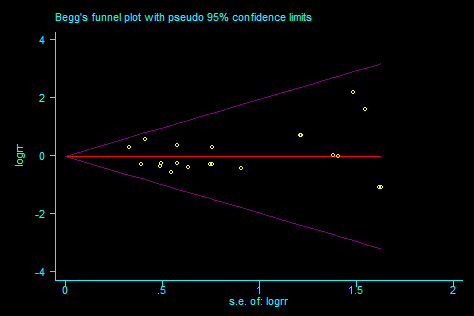

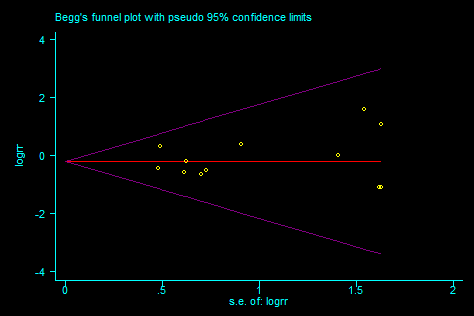


Any ST Definite ST


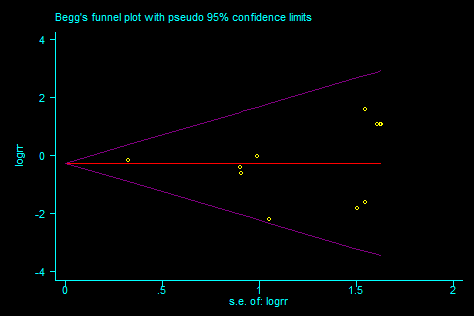

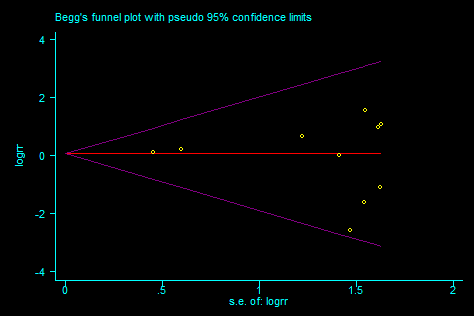


Early ST Late ST


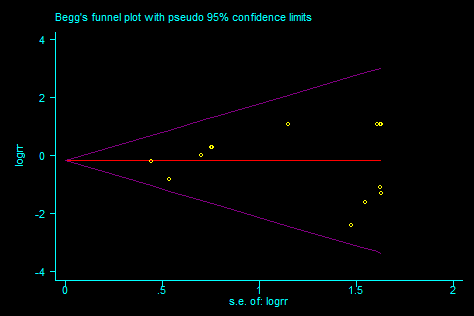

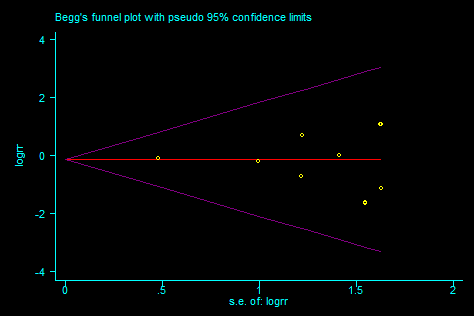


**RCTs OVER 1 year**

TLR MACE


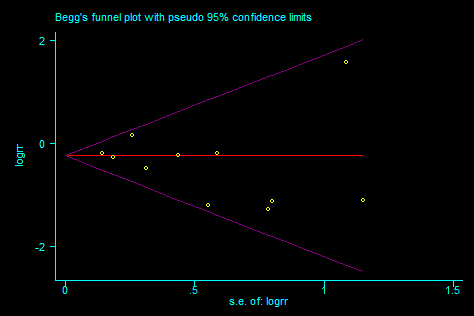

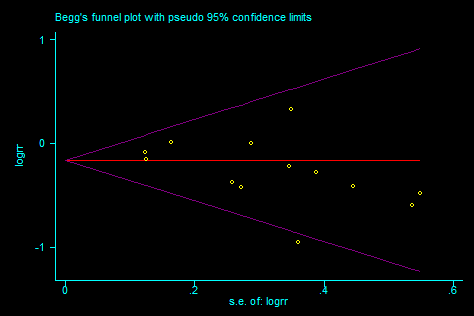


Myocardial infarction All-cause death


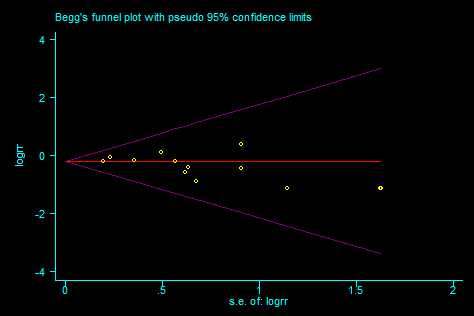

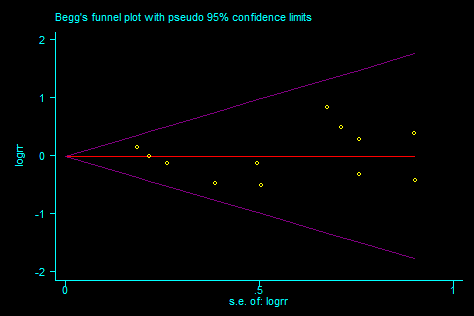


**RCT OVERALL**

TLR TVR


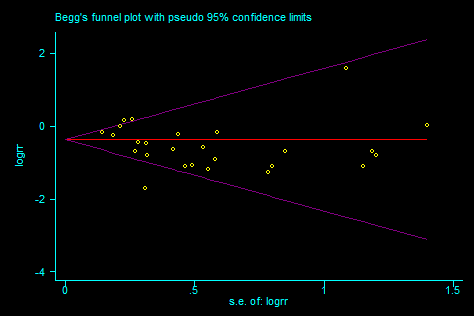

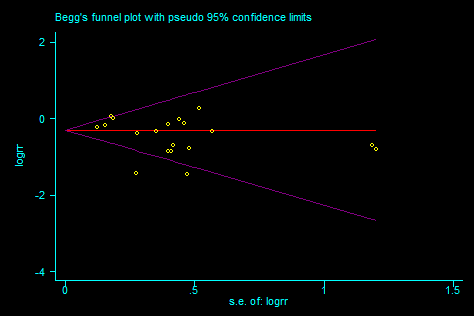


MACE Myocardial infarction


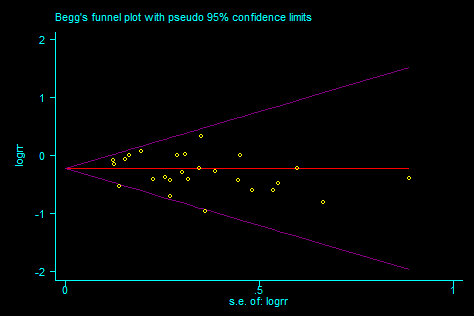

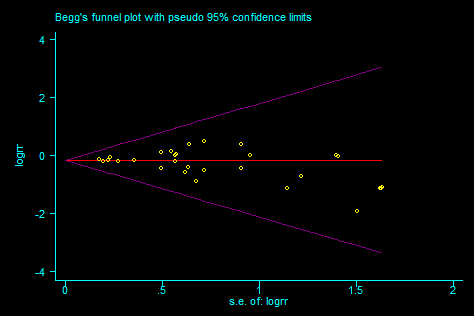


All-cause death Cardiac death


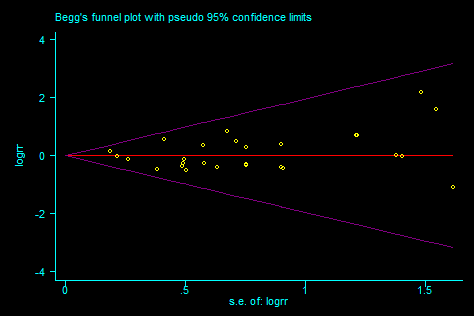

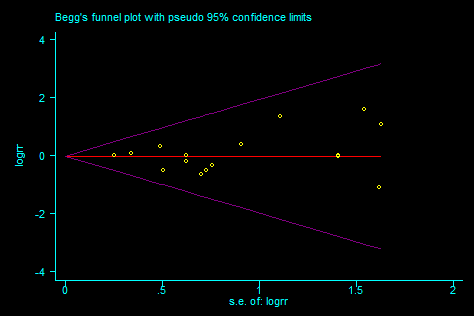


Any ST Definite ST


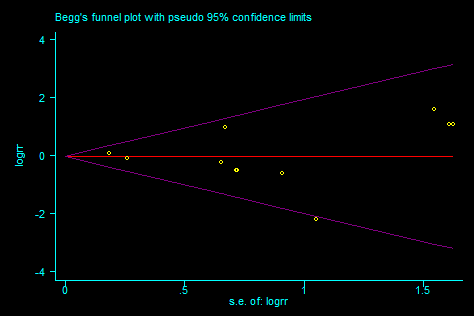

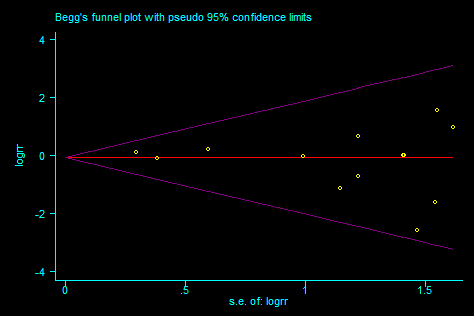


Definite + Probable ST


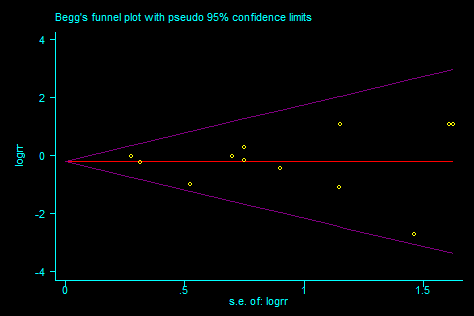


**Adjusted observational studies WITHIN 1 year**

MACE


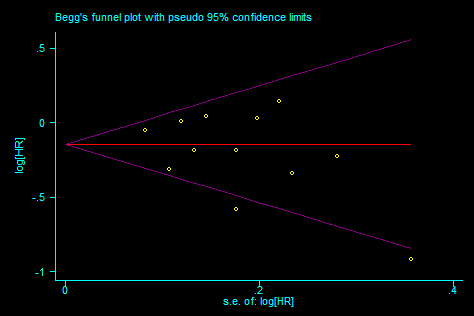


**Adjusted observational studies OVER 1 year**

MACE All-cause death


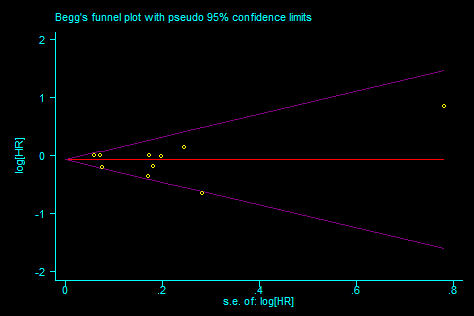

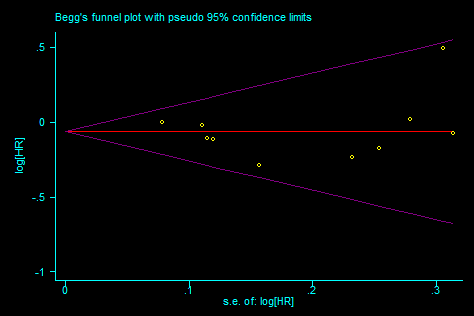


**Adjusted observational studies OVERALL**

TLR TVR


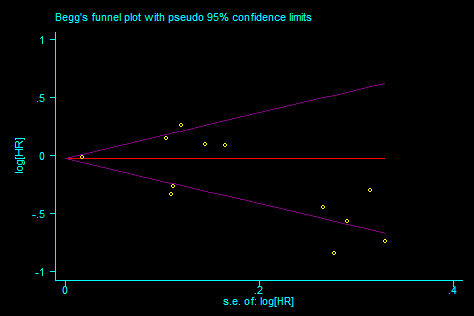

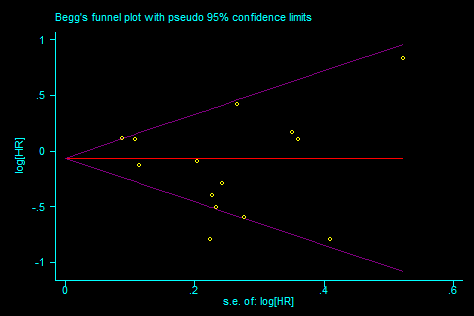


MACE All-cause death


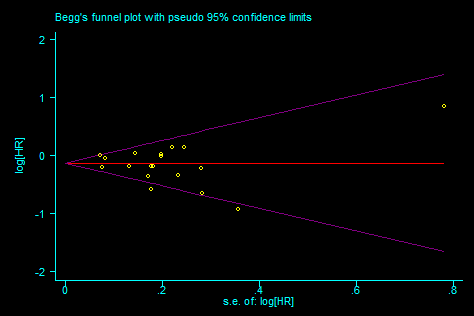

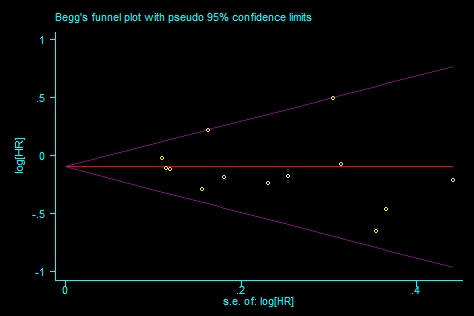


**Non-adjusted Observational studies WITHIN 1 year**

TLR TVR


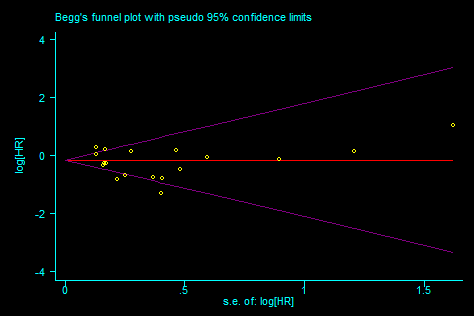

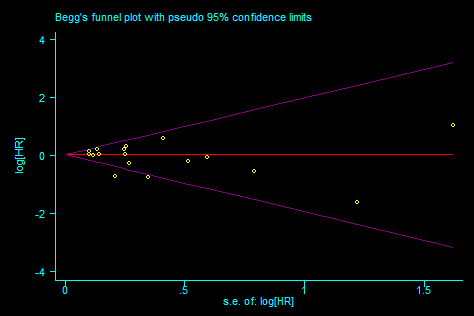


MACE Myocardial infarction


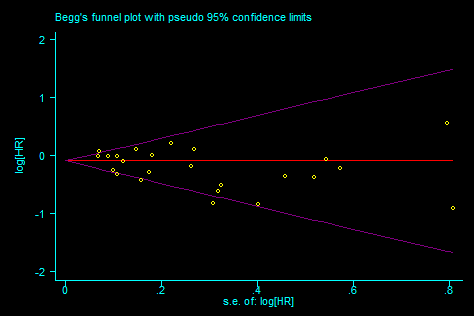

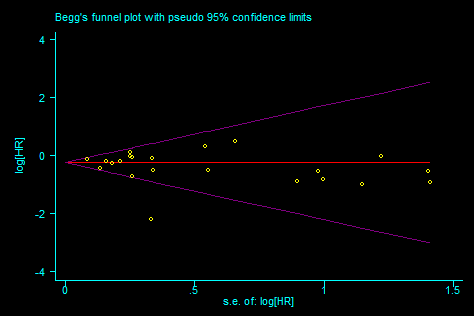


All-cause death Cardiac death


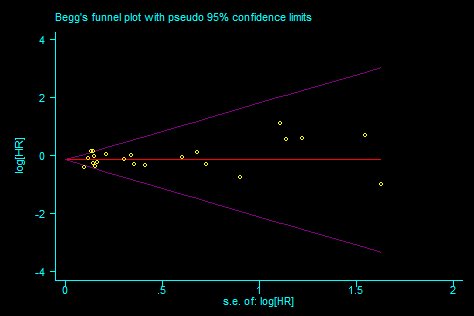

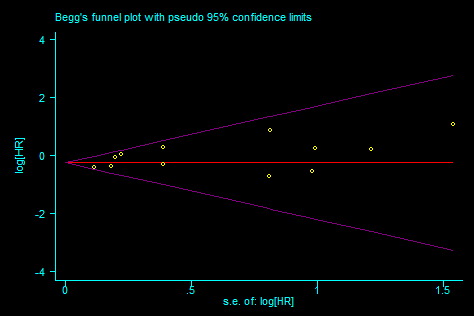


Any ST Definite + probable ST


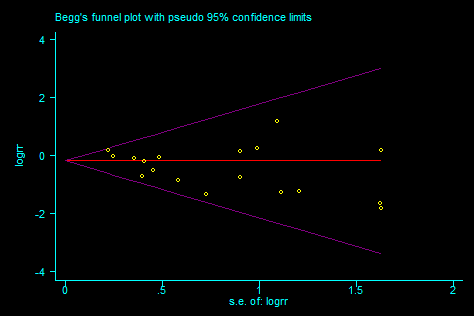

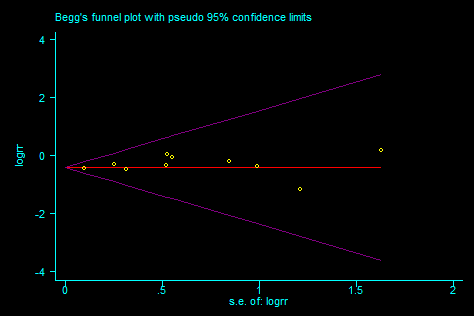


Early ST Late ST


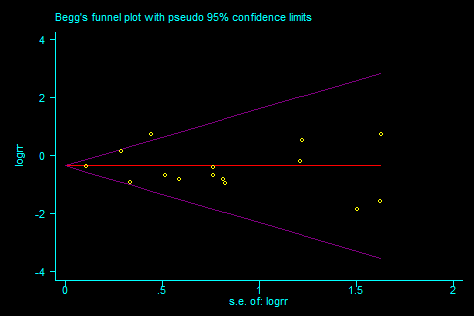

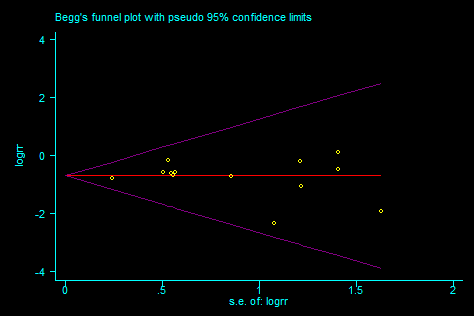


**Non-adjusted Observational studies OVER 1 year**

TLR TVR


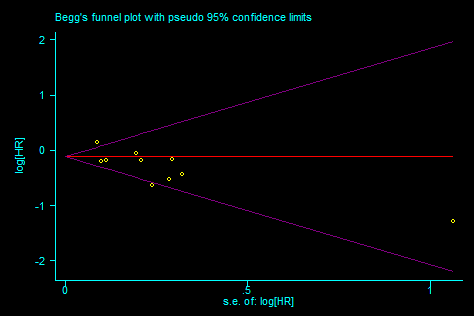

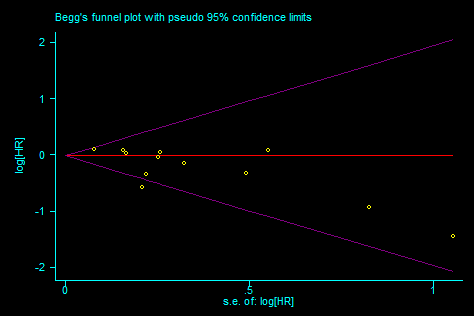


MACE Myocardial infarction


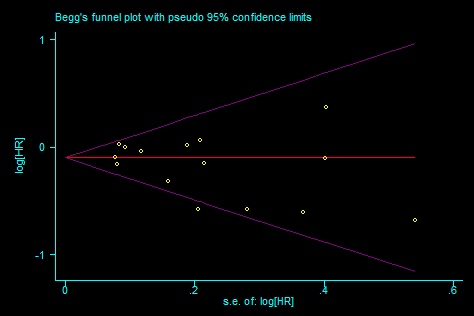

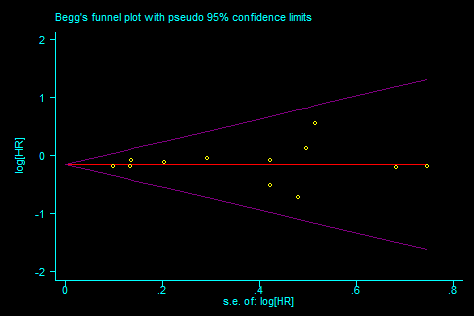


All-cause death Definite ST


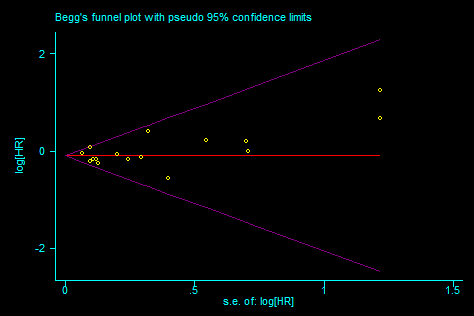

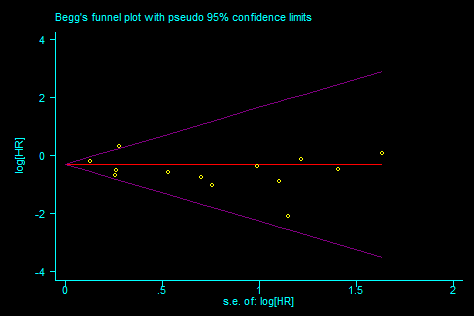


**Non-adjusted Observational studies OVERALL**

TLR TVR


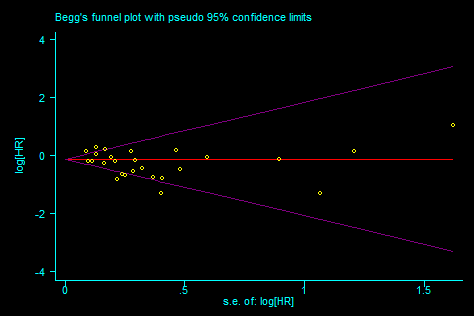

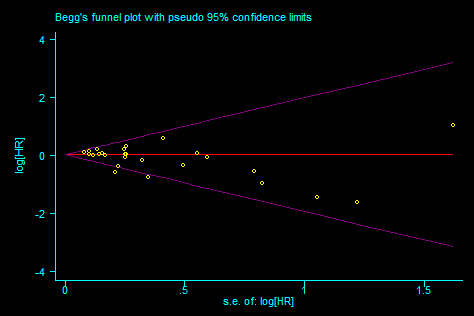


MACE Myocardial infarction


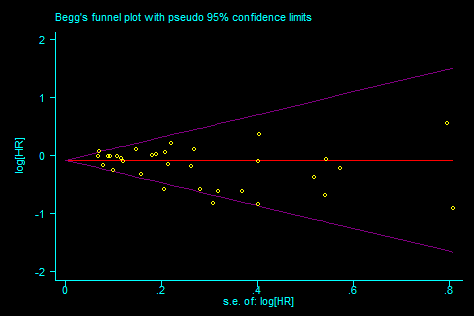

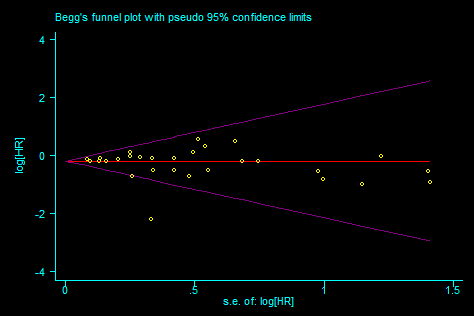


All-cause death Cardiac death


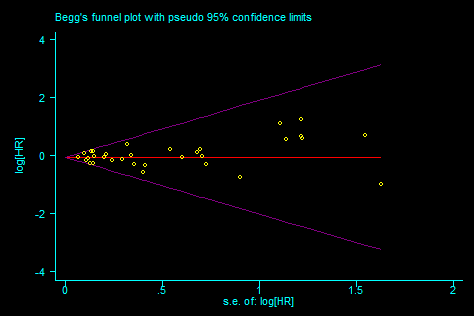

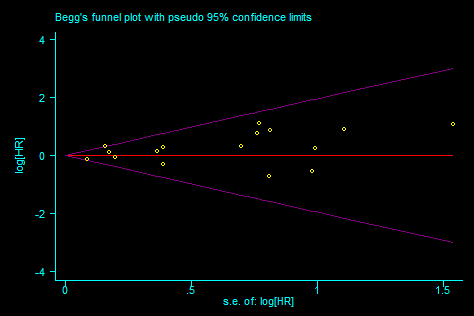


Any ST Definite ST


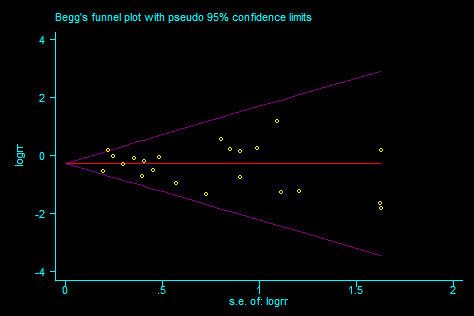

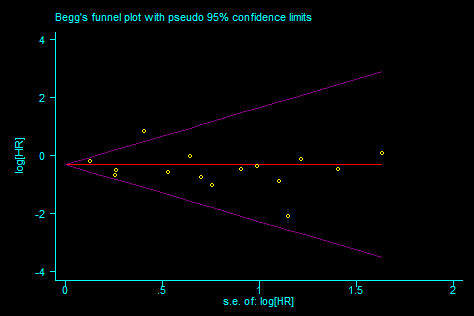


Definite + probable ST


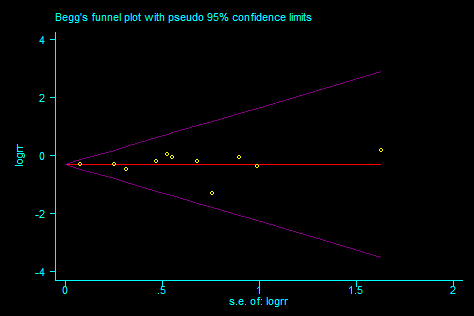

Supplement: File S5 — Funnel plots of the meta-analyses to detect publication bias. (DOC) [file pone.0097934.s005.doc]
