# Supplementary material for: Head-to-Head Comparison of Sirolimus-Eluting Stents versus Paclitaxel-Eluting Stents in Patients Undergoing Percutaneous Coronary Intervention: A Meta-Analysis of 76 Studies
Source: PLoS One. 2014 May 20;9(5):e97934. doi: 10.1371/journal.pone.0097934 (PMC4028235; doi:10.1371/journal.pone.0097934)
Supplement: Table S1 — Characteristics of studies included in the meta-analysis. (DOC) [file pone.0097934.s006.doc]

**Table S1.** Characteristics of studies included in the meta-analysis.

| Trial/Registry/  First Author | Year | patient profile | | Number of patients | | Age | | Men  % | | DM  % | | Hyper-  Tension% | | Hyperlipidaemia% | | Prior MI  % | | ACS  % | | FU. (m) |
| --- | --- | --- | --- | --- | --- | --- | --- | --- | --- | --- | --- | --- | --- | --- | --- | --- | --- | --- | --- | --- |
|  |  |  |  | S | P | S | P | S | P | S | P | S | P | S | P | S | P | S | P |  |
| Randomized Controlled Trials | | | | | | | | | | | | | | | | | | | | |
| BASKET  CORPAL | 2005  2005 | Unselected  Complex lesion | | 264  331 | 281  321 | 64±12  60±12 | 64±11  62±10 | 79  78 | 78  75 | 16  29 | 19  33 | 65 | 66 | 74 | 76 | 28 | 28 | 60 | 55 | 6 |
|  |  |  |  |  |  |  |  |  |  |  |  |  |  |  |  |  |  |  |  | 13 |
| ISAR-DESIRE | 2005 | ISR | | 100 | 100 | 63 | 65 | 78 | 79 | 31 | 27 | 51 | 56 | 58 | 53 | 45 | 55 |  |  | 12 |
| ISAR-Diabetes | 2005 | DM | | 125 | 125 | 68±10 | 68±10 | 75 | 71 |  |  | 56 | 66 | 58 | 62 | 31 | 39 | 45 | 34 | 9 |
| TAXi | 2005 | Unselected | | 102 | 100 | 65±10 | 63±10 | 79 | 83 | 33 | 36 | 60 | 63 | 77 | 79 | 33 | 29 | 18 | 14 | 6,36 |
| Cervinka *et al* | 2006 | Complex lesion | | 37 | 33 | 56±10 | 55±13 | 74 | 72 | 40 | 30 | 56 | 50 | 70 | 63 | 41 | 33 | 60 | 59 | 6 |
| Han *et al* | 2006 | Multi-vessel | | 202 | 196 |  |  |  |  |  |  |  |  |  |  |  |  |  |  | 19.5 |
| ISAR-SMART3 | 2006 | Small vessel | | 180 | 180 | 67±11 | 66±10 | 69 | 75 | 0 | 0 | 64 | 67 | 56 | 55 | 29 | 31 |  |  | 12 |
| Long DES II | 2006 | Long lesion | | 250 | 250 | 61±9 | 61±9 | 67 | 61 | 33 | 34 | 55 | 55 | 29 | 30 |  |  | 55 | 54 | 9 |
| REALITY | 2006 | Unselected | | 684 | 669 | 63±11 | 63±10 | 74 | 72 | 27 | 29 | 66 | 68 | 73 | 70 | 42 | 39 | 29 | 32 | 12 |
| Zhang *et al* | 2006 | Unselected | | 246 | 203 | 64±10 | 64±12 | 70 | 67 | 31 | 21 | 73 | 69 | 31 | 33 |  |  | 59 | 51 | 12 |
| Pan *et al* | 2007 | Bifurcation | | 103 | 102 | 62±11 | 61±10 | 81 | 83 | 34 | 40 | 58 | 62 | 54 | 46 | 19 | 16 | 59 | 61 | 24 |
| Petronio *et al* | 2007 | Long lesion | | 42 | 43 | 61±11 | 64±10 | 86 | 79 | 31 | 19 | 67 | 61 | 71 | 61 | 36 | 26 | 45 | 51 | 9 |
| DES-DIABETES | 2008 | DM | | 200 | 200 | 61±9 | 61±9 | 61 | 55 |  |  | 57 | 62 | 28 | 29 |  |  | 57 | 59 | 9,48 |
| Kim *et al* | 2008 | DM | | 85 | 84 | 63±8 | 62±9 | 72 | 76 |  |  | 65 | 73 | 31 | 31 | 15 | 14 | 65 | 58 | 6 |
| PROSIT | 2008 | STEMI | | 154 | 154 | 60±11 | 60±12 | 76 | 77 | 22 | 29 | 46 | 41 | 29 | 23 |  |  |  |  | 12,36 |
| SORTOUT II | 2008 | Unselected | | 1065 | 1033 | 65±12 | 64±11 | 74 | 76 | 15 | 15 |  |  | 58 | 60 | 26 | 25 | 52 | 49 | 18 |
| Tomai *et al* | 2008 | DM | | 60 | 60 | 65±10 |  | 73 |  |  |  | 82 |  |  |  | 43 |  | 53 |  | 8 |
| DiabeDES | 2009 | DM | | 76 | 77 | 66±8 | 65±10 | 84 | 74 | 83 | 87 | 63 | 75 |  |  |  |  | 33 | 33 | 8 |
| ISAR-LEFT-MAIN | 2009 | ULMCA | | 305 | 302 | 69±9 | 69±10 | 80 | 75 | 28 | 30 | 69 | 70 | 75 | 78 | 28 | 25 | 40 | 44 | 12,24 |
| Juwana *et al* | 2009 | STEMI | | 196 | 201 | 61±12 | 61±11 | 69 | 74 | 11 | 9 | 27 | 33 | 19 | 19 | 6 | 6 |  |  | 12 |
| Kim *et al* | 2009 | DM | | 85 | 84 |  |  |  |  |  |  |  |  |  |  |  |  |  |  | 36 |
| PASEO | 2009 | STEMI | | 90 | 90 | 62±16 | 63±16 | 71 | 69 | 28 | 23 | 28 | 27 |  |  | 16 | 14 |  |  | 12,48 |
| ZEST-AMI | 2009 | STEMI | | 110 | 110 | 58±11 | 59±11 | 86 | 83 | 26 | 24 | 38 | 54 | 41 | 46 |  |  |  |  | 12 |
| Hong *et al* | 2010 | DM | | 85 | 84 | 66±8 | 65±9 | 72 | 76 |  |  | 66 | 77 | 34 | 39 | 15 | 14 | 64 | 58 | 36 |
| ISAR-DESIRE2 | 2010 | ISR | | 225 | 225 | 66±11 | 67±10 | 79 | 74 | 38 | 34 | 72 | 72 | 67 | 65 | 45 | 44 | 20 | 17 | 12 |
| ODESSA | 2010 | Long lesion | | 22 | 22 | 67±10 | 67±9 | 68 | 82 | 36 | 18 | 50 | 50 | 36 | 50 | 46 | 23 | 46 | 50 | 12 |
| POET | 2010 | Single stent | | 108 | 125 |  |  |  |  |  |  |  |  |  |  |  |  |  |  | 9 |
| ZEST | 2010 | Unselected | | 878 | 884 | 62±10 | 62±10 | 67 | 66 | 28 | 28 | 59 | 61 | 51 | 51 | 4 | 5 | 56 | 55 | 12 |
| GARA-GARA | 2011 | Unselected | | 400 | 400 | 69±9 | 69±9 | 71 | 71 | 40 | 41 | 74 | 74 | 60 | 63 | 30 | 31 | 19 | 17 | 8 |
| KOMER | 2011 | STEMI | | 204 | 202 | 59±12 | 60±13 | 81 | 79 | 23 | 19 | 42 | 45 | 31 | 34 | 2 | 2 |  |  | 12,18 |
| Naples-Diabetes | 2011 | DM | | 76 | 75 | 64±8 | 64±10 | 57 | 59 |  |  | 73 | 76 | 62 | 64 | 43 | 35 | 16 | 17 | 8 |
| SIRTAX | 2011 | Unselected | | 503 | 509 | 62±11 | 62±12 | 76 | 78 | 22 | 18 | 60 | 62 | 61 | 57 | 29 | 30 | 51 | 52 | 12,60 |
| Adjusted Observational Studies | | | | | | | | | | | | | | | | | | | | |
| Hoye *et al* | 2005 | Bifurcation | | 144 | 104 | 62±11 | 60±12 | 74 | 73 | 19 | 17 | 43 | 46 | 57 | 63 | 35 | 39 | 35 | 44 | 6 |
| Iakovou *et al* | 2005 | Unselected | | 1062 | 1167 | 61±10 | 61±11 | 89 | 82 | 27 | 27 | 60 | 58 | 66 | 68 | 46 | 48 | 26 | 26 | 9 |
| T-SEARCH | 2005 | Unselected | | 508 | 576 | 61±11 | 62±11 | 68 | 74 | 18 | 18 | 41 | 42 | 56 | 62 | 30 | 35 | 55 | 55 | 12,72 |
| DEScover | 2006 | Unselected | | 3837 | 2636 | 64±12 | 65±12 | 68 | 68 | 33 | 31 | 76 | 76 | 76 | 77 | 27 | 28 | 53 | 55 | 12 |
| Kim *et al* | 2006 | Long lesion | | 184 | 166 | 62±9 | 62±10 | 71 | 74 | 31 | 32 | 57 | 56 | 27 | 31 |  |  | 46 | 50 | 9 |
| REAL | 2006 | Unselected | | 992 | 684 | 64±11 | 64±11 | 74 | 77 | 29 | 28 | 68 | 67 | 61 | 57 | 26 | 18 | 60 | 56 | 12,24 |
| Cosgrave *et al* | 2007 | Unselected | | 674 | 609 | 63±11 | 63±10 | 86 | 87 | 25 | 28 | 65 | 68 | 67 | 71 | 48 | 51 | 28 | 26 | 12 |
| Hannan *et al* | 2007 | Unselected | | 6914 | 4867 |  |  | 63 | 62 | 29 | 29 |  |  |  |  | 28 | 28 |  |  | 18 |
| STENT | 2007 | Unselected | | 4555 | 4671 | 63±12 | 63±12 | 66 | 65 | 31 | 31 | 74 | 74 | 68 | 70 | 23 | 23 | 78 | 81 | 9 |
| VERITAS | 2007 | Unselected | | 296 | 144 | 63±10 | 62±11 | 67 | 65 | 35 | 39 | 56 | 58 | 35 | 35 | 24 | 22 | 50 | 51 | 12 |
| Chieffo *et al* | 2008 | ULMCA | | 536 | 196 |  |  |  |  |  |  |  |  |  |  |  |  |  |  | 30 |
| KOMATE | 2008 | DM | | 428 | 206 | 63±10 | 65±9 | 60 | 65 |  |  | 69 | 68 | 37 | 35 | 10 | 11 | 58 | 52 | 36 |
| Choi *et al* | 2009 | AMI | | 56 | 53 | 62±12 | 63±12 | 79 | 62 | 27 | 38 | 48 | 59 | 73 | 62 | 9 | 6 |  |  | 6 |
| EVENT | 2009 | Unselected | | 3443 | 2592 | 64±12 | 65±11 | 68 | 69 | 35 | 35 | 79 | 79 | 77 | 73 | 34 | 34 | 32 | 39 | 12 |
| MAIN-COMPARE | 2009 | ULMCA | | 669 | 189 | 62±11 | 65±11 | 72 | 70 | 32 | 34 | 52 | 53 | 29 | 28 | 8 | 10 | 64 | 74 | 28 |
| TC-WYRE | 2009 | Unselected | | 742 | 816 | 64±12 | 65±11 | 69 | 65 | 33 | 35 |  |  |  |  | 43 | 40 | 28 | 28 | 12 |
| WDHR | 2009 | Unselected | | 2202 | 1298 | 62±11 | 62±11 | 71 | 74 | 17 | 17 | 39 | 41 | 48 | 52 | 23 | 22 | 49 | 52 | 12,36 |
| Bonello *et al* | 2010 | Small diameter | | 401 | 170 | 64±12 | 65±13 | 61 | 61 | 35 | 40 | 82 | 85 | 89 | 90 | 36 | 35 | 11 | 10 | 12 |
| COBIS | 2010 | Bifurcation | | 407 | 407 | 62±10 | 62±11 | 64 | 65 | 29 | 29 | 61 | 62 | 30 | 29 | 7 | 7 | 62 | 61 | 22 |
| DES.DE | 2010 | DM | | 612 | 914 | 67±10 | 67±10 | 75 | 69 |  |  | 94 | 92 | 83 | 83 | 33 | 31 | 39 | 42 | 12 |
| Ferenc *et al* | 2010 | Bifurcation | | 422 | 279 | 67±10 | 67±10 | 75 | 77 | 19 | 29 | 84 | 81 |  |  | 23 | 21 | 13 | 14 | 36 |
| REWARDS | 2010 | Unselected | | 2392 | 1119 | 63±12 | 66±12 | 63 | 67 | 36 | 36 | 82 | 81 | 88 | 84 | 33 | 36 | 62 | 71 | 12,36 |
| Ko *et al* | 2011 | Unselected | | 512 | 256 | 61±10 | 61±9 | 69 | 70 | 35 | 36 | 58 | 60 | 21 | 22 | 6 | 7 | 49 | 49 | 60 |
| Millauer *et al* | 2011 | Unselected | | 467 | 437 | 63±12 | 65±11 | 79 | 74 | 23 | 23 | 57 | 61 | 52 | 52 | 14 | 13 | 66 | 69 | 24 |
| Ishikawa *et al* | 2012 | Complex lesion | | 978 | 357 | 68±9 | 68±9 | 77 | 75 | 79 | 78 |  |  |  |  |  |  |  |  | 30 |
| KAMIR | 2012 | AMI+CKD | | 1845 | 1356 | 65±11 | 66±11 | 67 | 69 | 32 | 29 | 52 | 55 | 10 | 8 |  |  |  |  | 12 |
| Park *et al* | 2012 | Unselected | | 2810 | 2810 | 63±11 | 64±11 | 65 | 65 | 33 | 33 | 52 | 53 | 24 | 24 | 9 | 9 | 68 | 67 | 12,60 |
| Non-adjusted Observational Studies | | | | | | | | | | | | | | | | | | | | |
| Katritsis *et al* | 2006 | Unselected | | 139 | 156 | 58±11 | 58±11 | 91 | 85 | 26 | 19 | 71 | 56 | 84 | 78 |  |  | 44 | 49 | 13 |
| Qiao *et al* | 2006 | Complex lesion |  | |  |  |  |  |  |  |  |  |  |  |  |  |  |  |  | 6 |
| Stankovic *et al* | 2006 | DM | | 147 | 113 | 65±9 | 63±9 | 84 | 82 |  |  | 71 | 71 | 67 | 64 | 46 | 46 | 28 | 20 | 9 |
| Trabattoni *et al* | 2007 | Unselected | | 539 | 238 |  |  | 81 | 81 | 13 | 11 | 37 | 35 | 51 | 47 | 24 | 26 | 12 | 12 | 12 |
| Yang *et al* | 2007 | DM | | 101 | 63 | 59±11 | 60±10 | 68 | 70 |  |  | 68 | 67 | 39 | 40 |  |  | 36 | 44 | 12,36 |
| Chen *et al* | 2008 | Bifurcation | | 107 | 139 | 60±9 | 65±9 | 74 | 75 | 19 | 16 | 76 | 73 | 82 | 66 | 19 | 19 | 89 | 87 | 8 |
| Latib *et al* | 2008 | Bifurcation | | 161 | 112 | 64±11 | 62±11 | 83 | 88 | 38 | 32 | 71 | 71 | 62 | 71 | 73 | 48 | 52 | 36 | 35 |
| Hur *et al* | 2009 | Long lesion | | 77 | 45 | 66±9 | 66±9 | 62 | 49 | 34 | 31 | 51 | 44 | 39 | 40 | 12 | 9 | 69 | 73 | 12 |
| Chong *et al* | 2010 | DM | | 79 | 138 | 60±10 | 59±10 | 68 | 70 |  |  | 76 | 76 | 82 | 77 |  |  |  |  | 18 |
| Cicek *et al* | 2010 | Unselected | | 103 | 101 | 57±11 | 58±10 |  |  | 39 | 36 | 60 | 63 | 65 | 68 | 11 | 7 | 72 | 67 | 24 |
| Kim *et al* | 2010 | Single lesion | | 259 | 105 | 61±9 | 63±9 | 74 | 72 | 25 | 22 | 43 | 48 | 40 | 41 |  |  | 67 | 66 | 12 |
| Lee *et al* | 2010 | CAV | | 68 | 40 | 61±14 | 54±19 | 69 | 70 | 25 | 33 | 72 | 60 | 60 | 53 |  |  | 16 | 8 | 12 |
| Kataoka *et al* | 2011 | IGT | | 229 | 141 | 68±10 | 73±8 | 87 | 80 |  |  | 78 | 89 | 68 | 74 | 29 | 38 | 43 | 49 | 36 |
| Erdim *et al* | 2012 | STEMI | | 48 | 79 | 53±12 | 59±12 | 87 | 81 | 25 | 26 | 52 | 64 | 67 | 71 | 6 | 11 |  |  | 24 |
| Rabel *et al* | 2012 | Unselected | | 3819 | 4308 | 63±12 | 63±12 | 75 | 74 | 18 | 14 | 52 | 41 | 55 | 46 |  |  | 53 | 59 | 12,48 |
| Park *et al* | 2013 | Unselected | | 40 | 35 | 57±10 | 61±8 | 78 | 66 | 28 | 11 | 58 | 31 | 58 | 54 | 5 | 6 | 50 | 43 | 9,24 |

ACS: Acute coronary syndrome; AMI: Acute myocardial infarction; CAV: Cardiac Allograft Vasculopathy; CKD: Chronic kidney disease; DM: Diabetes mellitus; IGT: Impaired glucose tolerance; ISR: In stent restenosis; STEMI: ST-segment elevation myocardial infarction; ULMCA: Unprotected left main coronary artery disease.
