# Supplementary material for: Head-to-Head Comparison of Sirolimus-Eluting Stents versus Paclitaxel-Eluting Stents in Patients Undergoing Percutaneous Coronary Intervention: A Meta-Analysis of 76 Studies
Source: PLoS One. 2014 May 20;9(5):e97934. doi: 10.1371/journal.pone.0097934 (PMC4028235; doi:10.1371/journal.pone.0097934)
Supplement: Table S2 — Sensitivity analyses of outcomes in different subgroups. (DOC) [file pone.0097934.s007.doc]

**Table S2.** Sensitivity analyses of outcomes in different subgroups.

|  |  | | **MACE** | **Death** | **MI** | **TVR** | **TLR** | **Any ST** | **Definite ST** | **Def + Pro ST** |
| --- | --- | --- | --- | --- | --- | --- | --- | --- | --- | --- |
| Randomized Controlled Trials | | | | | | | | | | |
| Low bias study | | | | | | | | | | |
| 1 year | | Random | 0.76 (0.63, 0.92) | 0.96 (0.72, 1.29) | 0.89 (0.73, 1.08) | 0.56 (0.39, 0.80) | 0.55 (0.37, 0.80) | 0.76 (0.36, 1.61) | 0.92 (0.43, 1.94) | 0.75 (0.41, 1.38) |
|  |  | Fixed | 0.75 (0.66, 0.85) | 0.99 (0.75, 1.31) | 0.88 (0.73, 1.07) | 0.63 (0.53, 0.74) | 0.59 (0.49, 0.70) | 0.73 (0.44, 1.20) | 0.74 (0.37, 1.47) | 0.66 (0.41, 1.07) |
| > 1 year | | Random | 0.88 (0.75, 1.03) | 0.97 (0.76, 1.25) | 0.90 (0.65, 1.24) | 0.76 (0.62, 0.94) | 0.83 (0.65, 1.06) | 1.08 (0.77, 1.50) | 1.09 (0.64, 1.87) | 1.00 (0.62, 1.61) |
|  |  | Fixed | 0.87 (0.74, 1.02) | 0.97 (0.75, 1.24) | 0.90 (0.65, 1.23) | 0.75 (0.61, 0.93) | 0.82 (0.66, 1.03) | 1.09 (0.79, 1.51) | 1.09 (0.64, 1.86) | 1.01 (0.63, 1.61) |
| Overall | | Random | 0.82 (0.73, 0.92) | 0.98 (0.79, 1.21) | 0.90 (0.75, 1.08) | 0.64 (0.48, 0.85) | 0.61 (0.44, 0.85) | 0.97 (0.51, 1.83) | 1.00 (0.59, 1.68) | 0.79 (0.46, 1.35) |
|  |  | Fixed | 0.81 (0.73, 0.91) | 1.00 (0.81, 1.23) | 0.90 (0.75, 1.08) | 0.70 (0.60, 0.80) | 0.68 (0.58, 0.79) | 0.99 (0.73, 1.36) | 0.88 (0.54, 1.45) | 0.76 (0.51, 1.12) |
| DATP >6 m. | | | | | | | | | | |
| 1 year | Random | | 0.75 (0.64, 0.87) | 1.00 (0.76, 1.31) | 0.87 (0.72, 1.05) | 0.62 (0.47, 0.82) | 0.56 (0.41, 0.76) | 0.81 (0.49, 1.32) | 1.01 (0.56, 1.83) | 0.78 (0.44, 1.40) |
|  | Fixed | | 0.74 (0.66, 0.83) | 1.02 (0.78, 1.33) | 0.86 (0.72, 1.03) | 0.65 (0.56, 0.76) | 0.58 (0.49, 0.68) | 0.75 (0.48, 1.19) | 0.86 (0.50, 1.49) | 0.69 (0.43, 1.10) |
| > 1 year | Random | | 0.88 (0.77, 1.00) | 0.99 (0.81, 1.22) | 0.83 (0.65, 1.05) | 0.79 (0.66, 0.93) | 0.77 (0.61, 0.98) | 1.01 (0.77, 1.33) | 0.98 (0.65, 1.49) | 0.90 (0.62, 1.31) |
|  | Fixed | | 0.87 (0.77, 0.99) | 0.99 (0.81, 1.22) | 0.82 (0.65, 1.04) | 0.79 (0.66, 0.93) | 0.78 (0.66, 0.94) | 1.02 (0.78, 1.33) | 0.97 (0.64, 1.47) | 0.91 (0.63, 1.31) |
| Overall | Random | | 0.82 (0.74, 0.90) | 1.01 (0.84, 1.21) | 0.86 (0.73, 1.00) | 0.71 (0.58, 0.86) | 0.62 (0.49, 0.78) | 0.97 (0.73, 1.29) | 0.95 (0.65, 1.39) | 0.82 (0.59, 1.15) |
|  | Fixed | | 0.85 (0.73, 1.00) | 1.02 (0.86, 1.22) | 0.85 (0.73, 1.00) | 0.73 (0.65, 0.83) | 0.67 (0.58, 0.76) | 0.96 (0.74, 1.24) | 0.88 (0.61, 1.27) | 0.78 (0.56, 1.07) |
| Adjusted Observational Studies | | | | | | | | | | |
| DATP >6 m. | | | | | | | | | | |
| 1 year | Random | | 0.75 (0.63, 0.90) | 0.77 (0.64, 0.93) | 0.70 (0.45, 1.09) | 0.55 (0.38, 0.79) | 0.73 (0.50, 1.06) |  | 0.76 (0.47, 1.24) |  |
|  | Fixed | | 0.78 (0.70, 0.87) | 0.77 (0.64, 0.93) | 0.73 (0.55, 0.98) | 0.55 (0.38, 0.79) | 0.78 (0.64, 0.96) |  | 0.73 (0.54, 0.98) |  |
| > 1 year | Random | | 0.91 (0.82, 1.02) | 0.93 (0.85, 1.02) | 0.85 (0.72, 1.01) | 0.85 (0.67, 1.09) | 0.82 (0.67, 1.00) |  | 0.52 (0.39, 0.68) |  |
|  | Fixed | | 0.94 (0.87, 1.00) | 0.93 (0.85, 1.02) | 0.85 (0.72, 1.01) | 0.93 (0.82, 1.07) | 0.98 (0.94, 1.01) |  | 0.52 (0.39, 0.68) |  |
| Overall | Random | | 0.80 (0.71, 0.92) | 0.86 (0.77, 0.97) | 0.84 (0.71, 0.99) | 0.65 (0.52, 0.81) | 0.80 (0.67, 0.96) |  | 0.57 (0.40, 0.82) |  |
|  | Fixed | | 0.85 (0.78, 0.92) | 0.86 (0.77, 0.97) | 0.84 (0.71, 0.99) | 0.64 (0.53, 0.78) | 0.97 (0.94, 1.01) |  | 0.60 (0.46, 0.78) |  |
| Non-adjusted Observational Studies | | | | | | | | | | |
| DATP >6 m. | | | | | | | | | | |
| 1 year | Random | | 0.83 (0.75, 0.93) | 0.80 (0.72, 0.88) | 0.81 (0.72, 0.90) | 0.91 (0.72, 1.15) | 0.78 (0.63, 0.98) | 0.63 (0.46, 0.87) | 0.76 (0.45, 1.27) | 0.67 (0.56, 0.80) |
|  | Fixed | | 0.87 (0.81, 0.93) | 0.80 (0.72, 0.88) | 0.81 (0.72, 0.90) | 0.98 (0.85, 1.12) | 0.88 (0.79, 0.98) | 0.63 (0.46, 0.85) | 0.71 (0.54, 0.92) | 0.67 (0.56, 0.80) |
| > 1 year | Random | | 0.92 (0.85, 0.99) | 0.88 (0.81, 0.95) | 0.86 (0.76, 0.97) | 0.94 (0.80, 1.11) | 0.82 (0.72, 0.92) |  | 0.73 (0.59, 0.90) |  |
|  | Fixed | | 0.92 (0.86, 0.99) | 0.88 (0.81, 0.95) | 0.86 (0.76, 0.97) | 0.94 (0.80, 1.11) | 0.82 (0.72, 0.92) |  | 0.75 (0.62, 0.90) |  |
| Overall | Random | | 0.90 (0.83, 0.97) | 0.90 (0.83, 0.97) | 0.87 (0.79, 0.95) | 1.04 (0.92, 1.18) | 0.78 (0.66, 0.92) | 0.65 (0.52, 0.82) | 0.70 (0.53, 0.93) | 0.75 (0.65, 0.87) |
|  | Fixed | | 0.91 (0.86, 0.96) | 0.90 (0.83, 0.97) | 0.87 (0.79, 0.95) | 1.04 (0.92, 1.18) | 0.84 (0.77, 0.92) | 0.65 (0.52, 0.81) | 0.74 (0.61, 0.89) | 0.75 (0.65, 0.87) |

ACS: Acute coronary syndrome; DATP: Dual antiplatelet therapy; Def + Pro ST: Definite and probable stent thrombosis; MACE: Major adverse cardiac event; MI: Myocardial infarction; ST: Stent thrombosis; TLR: Target lesion revascularization; TVR: Target vessel revascularization.
