# Supplementary material for: Head-to-Head Comparison of Sirolimus-Eluting Stents versus Paclitaxel-Eluting Stents in Patients Undergoing Percutaneous Coronary Intervention: A Meta-Analysis of 76 Studies
Source: PLoS One. 2014 May 20;9(5):e97934. doi: 10.1371/journal.pone.0097934 (PMC4028235; doi:10.1371/journal.pone.0097934)
Supplement: Table S3 — Sensitivity analyses of angiographic outcomes of different subgroups in randomized controlled trials. (DOC) [file pone.0097934.s008.doc]

**Table S3.** Sensitivity analyses of angiographic outcomes of different subgroups in randomized controlled trials.

| **Subgroup** | **Model** | **In-stent Restenosis** | **In-segment Restenosis** | **In-stent LLL** | **In-segment LLL** |
| --- | --- | --- | --- | --- | --- |
| Low bias study | Random | 0.37 (0.23, 0.58) | 0.47 (0.32, 0.69) | -0.20 (-0.25, -0.14) | -0.20 (-0.25, -0.14) |
|  | Fixed | 0.39 (0.33, 0.47) | 0.54 (0.47, 0.62) | -0.18 (-0.20, -0.16) | -0.18 (-0.20, -0.16) |
| DATP >6 m. | Random | 0.42 (0.28, 0.62) | 0.50 (0.37, 0.67) | -0.40 (-0.49, -0.30) | -0.40 (-0.49, -0.30) |
|  | Fixed | 0.41 (0.34, 0.49) | 0.54 (0.48, 0.61) | -0.38 (-0.42, -0.34) | -0.38 (-0.42, -0.34) |
| Non-ACS study | Random | 0.72 (0.56, 0.91) | 0.72 (0.59, 0.90) | -0.15 (-0.22, -0.08) | -0.15 (-0.22, -0.08) |
|  | Fixed | 0.71 (0.56, 0.90) | 0.77 (0.66, 0.89) | -0.13 (-0.16, -0.10) | -0.13 (-0.16, -0.10) |

ACS: Acute coronary syndrome; DATP: Dual antiplatelet therapy; LLL: Late lumen loss.
